# Contribution of spinal cord glial cells to *L. amazonensis* experimental infection-induced pain in BALB/c mice

**DOI:** 10.1186/s12974-019-1496-2

**Published:** 2019-05-28

**Authors:** Sergio M. Borghi, Victor Fattori, Felipe A. Pinho-Ribeiro, Talita P. Domiciano, Milena M. Miranda-Sapla, Tiago H. Zaninelli, Rubia Casagrande, Phileno Pinge-Filho, Wander R. Pavanelli, Jose C. Alves-Filho, Fernando Q. Cunha, Thiago M. Cunha, Waldiceu A. Verri

**Affiliations:** 1 Departament of Pathology, Biological Sciences Center, Londrina State University, Rodovia Celso Garcia Cid, Pr 445, Km 380 Cx. Postal 10.011, Londrina, Paraná CEP 86057-970 Brazil; 2 Center for Research in Health Sciences, University of Northern Paraná - Unopar, Rua Marselha, 591, Jardim Piza, Londrina, Paraná 86041-140 Brazil; 3 Departament of Pharmaceutical Sciences, Health Sciences Center, University Hospital, Londrina State University, Avenida Robert Koch, 60, Londrina, Paraná 86038-350 Brazil; 4 Department of Pharmacology, Ribeirão Preto Medical School, University of São Paulo, Avenida Bandeirantes, 3900, Ribeirão Preto, São Paulo 14049-900 Brazil

**Keywords:** *L. amazonensis*, Hyperalgesia, Astrocytes, Microglia, NFκB

## Abstract

**Background:**

The cellular and molecular pathophysiological mecha\nisms of pain processing in neglected parasitic infections such as leishmaniasis remain unknown. The present study evaluated the participation of spinal cord glial cells in the pathophysiology of pain induced by *Leishmania amazonensis* infection in BALB/c mice.

**Methods:**

Mice received intra-plantar (i.pl.) injection of *L. amazonensis* (1 × 10^5^) and hyperalgesia, and paw edema were evaluated bilaterally for 40 days. The levels of TNF-α and IL-1β, MPO activity, and histopathology were assessed on the 40th day. ATF3 mRNA expression was assessed in DRG cells at the 30th day post-infection. Blood TNF-α and IL-1β levels and systemic parasite burden were evaluated 5–40 days after the infection. At the 30th day post-infection *L. amazonensis*, the effects of intrathecal (i.t.) treatments with neutralizing antibody anti-CX_3_CL1, etanercept (soluble TNFR2 receptor), and interleukin-1 receptor antagonist (IL-1ra) on infection-induced hyperalgesia and paw edema were assessed. In another set of experiments, we performed a time course analysis of spinal cord GFAP and Iba-1 (astrocytes and microglia markers, respectively) and used confocal immunofluorescence and Western blot to confirm the expression at the protein level. Selective astrocyte (α-aminoadipate) and microglia (minocycline) inhibitors were injected i.t. to determine the contribution of these cells to hyperalgesia and paw edema. The effects of i.t. treatments with glial and NFκB (PDTC) inhibitors on spinal glial activation, TNF-α, IL-1β, CX_3_CR1 and CX_3_CL1 mRNA expression, and NFκB activation were also evaluated. Finally, the contribution of TNF-α and IL-1β to CX_3_CL1 mRNA expression was investigated.

**Results:**

*L. amazonensis* infection induced chronic mechanical and thermal hyperalgesia and paw edema in the infected paw. Mechanical hyperalgesia was also observed in the contralateral paw. TNF-α, IL-1β, MPO activity, and epidermal/dermal thickness increased in the infected paw, which confirmed the peripheral inflammation at the primary foci of this infection. ATF3 mRNA expression at the ipsilateral DRG of the infected paw was unaltered 30 days post-infection. TNF-α and IL-1β blood levels were not changed over the time course of disease, and parasitism increased in a time-dependent manner in the ipsilateral draining lymph node. Treatments targeting CX_3_CL1, TNF-α, and IL-1β inhibited *L. amazonensis*-induced ongoing mechanical and thermal hyperalgesia, but not paw edema. A time course of GFAP, Iba-1, and CX_3_CR1 mRNA expression indicated spinal activation of astrocytes and microglia, which was confirmed at the GFAP and Iba-1 protein level at the peak of mRNA expression (30th day). Selective astrocyte and microglia inhibition diminished infection-induced ipsilateral mechanical hyperalgesia and thermal hyperalgesia, and contralateral mechanical hyperalgesia, but not ipsilateral paw edema. Targeting astrocytes, microglia and NFκB diminished *L. amazonensis*-induced GFAP, Iba-1, TNF-α, IL-1β, CX_3_CR1 and CX_3_CL1 mRNA expression, and NFκB activation in the spinal cord at the peak of spinal cord glial cells activation. CX_3_CL1 mRNA expression was also detected in the ipsilateral DRG of infected mice at the 30th day post-infection, and the i.t. injection of TNF-α or IL-1β in naïve animals induced CX_3_CL1 mRNA expression in the spinal cord and ipsilateral DRG.

**Conclusions:**

*L. amazonensis* skin infection produces chronic pain by central mechanisms involving spinal cord astrocytes and microglia-related production of cytokines and chemokines, and NFκB activation contributes to *L. amazonensis* infection-induced hyperalgesia and neuroinflammation.

**Electronic supplementary material:**

The online version of this article (10.1186/s12974-019-1496-2) contains supplementary material, which is available to authorized users.

## Background

Leishmaniasis represents a group of neglected diseases caused by the protozoan parasites from *Leishmania* genus. The anthroponotic cutaneous leishmaniasis (CL) is the main form of the disease in humans [1] and is characterized by the development of large cutaneous wounds and scars. This disease causes significant morbidity and is often associated with aesthetic-induced social dislocation and functional disorders [1, 2]. Despite the general assumption that skin wounds caused by leishmaniasis are painless, a growing body of evidence from pre-clinical [1–4] and clinical studies [1, 5–11] suggests that pain may be a neglected symptom in leishmaniasis. This evidence rises up the challenge of understanding the pain and painless mechanisms of leishmaniasis. In this sense, pre-clinical studies focusing on the pathophysiology of *Leishmania*-induced pain are strongly encouraged.

Evidence shows that peripheral *Leishmania* (*L. major*) infection causes pain in mice, whereas the higher *L. major* load the higher and chronic hyperalgesia [12]. *L. major* peripheral infection drives an immune response in the site of parasite inoculation culminating in an inflammatory response characterized by the production of cytokines and growth factors [3, 12, 13] with recognized pro-hyperalgesic function [14, 15]. These molecules can both activate and sensitize the primary nociceptor neurons, which make synapse with spinal cord neurons that transmit the peripheral nociceptive information to the brain [14, 15]. The spinal cord is an important structure where the transmission of peripheral inputs to the cortex can be either suppressed or exacerbated by tissue resident cells [14, 15]. Recent data demonstrated that the pro-inflammatory and hyperalgesic cytokine tumor necrosis factor alpha (TNF-α) and the transcription factor nuclear factor kappa B (NFκB) synergize to maintain the *L. amazonensis* infection-driven hyperalgesic state in the spinal cord [2], which supports the role of spinal cord neuroinflammation in leishmaniasis-induced pain.

Spinal cord glial cells constitute important sentinels to detect physiological and pathological changes in the central nervous system. In response to peripheral stimuli, these cells can respond by releasing mediators that activate and sensitize the peripheral primary nociceptive neurons. Via neuronal release of CX3CL1, the nociceptive input is transmitted to the spinal cord glial cells, which became activated and release mediators such as cytokines, chemokines, neurotrophic factors, and prostanoids that trigger neuroinflammation and central pain sensitization mechanisms [15]. This pathological mechanism is observed in inflammatory, neuropathic, and cancer pain models and involves neural plasticity that ultimately sensitizes the peripheral and central nervous system (CNS) [15–19]. However, whether spinal cord astrocytes and microglia represent key cellular components in *Leishmania* spp.-induced hyperalgesia in BALB/c mice remains to be determined, and therefore, it was the aim of the present study.

## Methods

### Animals

The experiments were conducted only on health immunocompetent male BALB/c mice, a prototype strain of susceptibility to *Leishmania* infection, weighing between 20 and 25 g, 4–6 weeks old, obtained from Fundação Oswaldo Cruz (FIOCRUZ), Paraná State, Brazil, and from State University of Londrina (UEL), Paraná State, Brazil. The selective use of male mice considered the gender dimorphism in pain regulation in this species [20, 21]. The use of BALB/c in models of leishmaniasis is supported by literature showing host genetic background influences the outcomes and the severity of the disease. BALB/c is a mouse strain that is highly susceptible to *Leishmania* spp*.* infection compared to other inbred mice such as the relative resistant C57BL/6 and is often used to study pain mechanisms, immunopathology, and therapeutic approaches in experimental leishmaniasis [22–25]. Mice were carefully kept under pathogen-free conditions in cages with individual ventilation in a rack system designed for mouse housing with regular shaving bedding, five animals per cage (32 × 20 × 21 cm, with 12.7 cm of internal height and 451 cm^2^ of floor area) (Alesco Indústria e Comércio LTDA, Monte Mor, São Paulo, Brazil), housed in standard clear plastic cages with free access to water and food (based on maize, Nuvilab CR-1 commercial food, Quimtia SA, Colombo, Paraná, Brazil), light/dark cycle of 12/12 h, exhausted air and controlled temperature (22 ± 2 °C), and were maintained in the vivarium of the Department of Pathology of State University of Londrina for at least 1 week before the experiments. Mice were used only once per experiment. For behavioral assessments, after the transport to the laboratory from the animal care facility, animals were acclimatized to the testing room at least 1 h before the experiments, which were conducted during the light cycle. For euthanasia, at the end of experiments, mice were anesthetized with isoflurane 5% (Abbott Park, IL, USA) and terminally killed by cervical dislocation followed by decapitation always during the light cycle in the laboratory. The mice were continuously monitored regarding welfare-related assessment before, during, and after the experiments. Clinical signs such as body weight, erection of the back hairs (which occur when the animals are irritated or alarmed), diarrhea, lethargy, and paralysis were also recorded. Mice that present clinical signs of severe disease before the end of the experimental protocol were immediately euthanized by cervical dislocation, following the guidelines of the Ethics Committee on Animal Use (CEUA) of UEL.

### Ethics statement

All animals were used according to the protocols approved by the CEUA (the Animal Welfare Ethical Review Board) of the State University of Londrina, registered under the number 1067.2015.64. Animals’ care and handling procedures were carried out following the Brazilian Council on Animal Experimentation (CONCEA), the Directive 2010/63/EU for animal experiments, and in accordance with the International Association for Study of Pain (IASP) guidelines. All efforts were made to minimize the number of animals used and their suffering.

### *L. amazonensis* promastigotes culture and experimental infection

Promastigotes forms of *L. (L.) amazonensis* (MHOM/BR/1989/166MJO; isolated and characterized from human patient in the city of Maringá, Paraná, Brazil) in the stationary growth phase were obtained from homogenate of popliteal lymph nodes of infected BALB/c mice. The division of promastigote forms were cultured in 199 medium (Invitrogen-GIBCO) supplemented with 10% fetal bovine serum, 1 M Hepes, 0.1% L-glutamine, 1% penicillin-streptomycin solution, 10% sodium bicarbonate, and 1% human urine. Cultures were incubated in a BOD-type incubator at 25 °C in 25-cm^2^ flasks. In our laboratory, the use of 199 medium is well established as promastigote culture medium and follows the formulation and chemical composition defined previously. Mice used in the present study were infected subcutaneously in the plantar region of the right hind paw with *L. amazonensis* promastigote forms (1 × 10^5^/20 μL) [2, 25]. All procedures related to *L. amazonensis* manipulations and infection protocols were conducted under Biosafety Level 2 protocols and guidelines.

### Drugs and administration

The following materials were obtained from the sources indicated. Saline solution 0.9% was obtained from Gaspar Viana S/A (Fortaleza, CE, Brazil). Ketamin and xylazine where obtained from Syntec do Brazil (Santana de Parnaíba, SP, Brazil). Dimethyl sulfoxide (DMSO), ethylenediamine tetra acetic acid (EDTA), Tween 80, α-aminoadipate, minocycline, and pyrrolidine dithiocarbamate (PDTC) were obtained from Sigma-Aldrich (St. Louis, MO, USA). Mouse neutralizing antibody anti-C-X_3_-C motif chemokine ligand 1 (CX_3_CL1, also known as fractalkine; AF472) (0.25–2.5 μg/5 μL in saline) and normal isotype-matched antibody (control IgG, 1 μL/4 μL in saline; AB-108-C) were obtained from R&D Systems (Minneapolis, MN, USA). Etanercept [Enbrel®, soluble tumor necrosis factor receptor 2 (TNFR2), 3–10 ng/5 μL in saline] was obtained from Wyeth (São Paulo, SP, Brazil). Interleukin-1 receptor antagonist (IL-1ra, 30–100 pg/5 μL in saline) was obtained from the National Institute for Biological Standards and Control (NIBSC, South Mimms, Hertfordshire, UK). Mouse recombinant TNF-α (1 ng/5 μL in saline) and IL-1β (1 ng/5 μL in saline) were obtained from eBioscience (San Diego, CA, USA). α-Aminoadipate (selective astrocyte inhibitor, 30–100 nmol/5 μL in saline), minocycline (microglia inhibitor, 50–150 μg/5 μL in saline), and pyrrolidine dithiocarbamate (PDTC; NFκB inhibitor, 300 μg/5 μL in saline) were diluted immediately before use in 2% DMSO + 2% Tween 80 + 96% saline, 2% DMSO + 98% saline, and 20% DMSO + 80 % saline, respectively. Saline was used as vehicle (5 μL) for treated groups described above with the exception of negative control group of anti-CX_3_CL1 antibody treatment that received an isotype-matched antibody injection at the same concentration of anti-CX_3_CL1 antibody (IgG, 5 μL). Selected doses used in the present study were defined considering its analgesic effects demonstrated earlier. Treatments were performed by intrathecal (i.t.) route to achieve a local effect on spinal sites. Drug administration was performed in unconscious animals (lumbar segment, L_4_–L_6_ zone) under anesthesia with isoflurane 5%, which was selected since it allows anesthesia during a brief period by inhalation (Abbott Park, IL, USA).

### General experimental procedures

In the first set of experiments, mice (*n* = 12) were initially divided into control non-infected (*n* = 6) and infected groups (*n* = 6) to evaluate mechanical hyperalgesia, thermal hyperalgesia, and paw edema (bilaterally in infected animals) during 40 days. At the end of this period (day 40), samples of control non-infected (right paw) and ipsilateral (right paw) and contralateral (left paw) paw tissues of infected animals were collected for the determination of tumor necrosis factor-α (TNF-α) and interleukin-1 beta (IL-1β) production and myeloperoxidase (MPO) activity. Additionally, representative images of control non-infected paw and ipsilateral and contralateral paw of infected mice together with the histological images and score of all experimental groups were provided at day 40 after the infection. Subsequently, the mRNA expression of cyclic AMP-dependent transcription factor 3 (ATF3) in the dorsal root ganglia (DRG) cells was determined in control non-infected animals and bilaterally in infected animals also at 30th day post-infection. Samples of ipsilateral DRG of chronic constriction injury (CCI) positive control group were included for comparison with the group infected with *L. amazonensis* (*n* = 18; *n* = 6 for non-infected group, *n* = 6 for infected group, and *n* = 6 for CCI group). The temporal profile of blood TNF-α and IL-1β levels in comparison with control group (5–40 days post-infection) as well as parasitism in ipsilateral draining lymph node, spleen, and contralateral lymph node (0–40 days post-infection) were evaluated (*n* = 36; *n* = 6 per group). Next, in another set of experiment (*n* = 30), i.t. treatments targeting CX_3_CL1, TNF-α, and IL-1β with neutralizing anti-CX_3_CL1 antibody (*n* = 6), etanercept (*n* = 6), and IL-1ra (*n* = 6), respectively, were performed at the 30th day post-infection to evaluate their effects upon *L. amazonensis*-induced mechanical hyperalgesia, thermal hyperalgesia, and paw edema in comparison with vehicle-treated control (*n* = 6) and non-infected group (*n* = 6) for up to 96 h after the treatments to determine the duration of pharmacological activity. In a second round of experiments, the temporal profile (5–40 days post-infection) of mRNA expression of glial fibrillary acidic protein (GFAP), ionized calcium-binding adapter molecule 1 (Iba-1), and C-X_3_-C motif chemokine receptor 1 (CX_3_CR1) in the spinal cord of infected animals (*n* = 30; *n* = 6 per day) were evaluated in comparison with control non-infected group (*n* = 6) to determine the peak of their expression during the course of the experimental leishmaniasis. After detecting the peak of mRNA expression of GFAP and Iba-1 in control non-infected and infected mice, immunofluorescence of bilateral dorsal horn and in more detail only in ipsilateral side of the infected paw (*n* = 8; *n* = 4 for non-infected group and *n* = 4 for infected group) and Western blot (whole L_4_–L_6_ spinal segment, *n* = 6; *n* = 3 for non-infected group and *n* = 3 for infected group) assays were performed in spinal cord samples collected at the day of the peak of mRNA expression (30th day). Nuclear factor of kappa light polypeptide gene enhancer in B cells inhibitor alpha (IκB-α) protein levels were also determined by Western blot assay in infected (*n* = 3) and non-infected (*n* = 3) mice. Subsequently, at the 30th day post-infection, mice were treated by i.t. route with the astrocyte inhibitor α-aminoadipate and microglia inhibitor minocycline to evaluate the role of spinal cord glial cells in ongoing leishmaniasis-induced ipsilateral mechanical hyperalgesia, thermal hyperalgesia, and paw edema in infected paws in comparison with vehicle-treated and control non-infected group for up to 96 h after the treatments to determine the duration of pharmacological activity. For this purpose, mice (*n* = 48) were divided into a total of six groups according to the inhibitor and administrated dose, namely, control non-infected (*n* = 12), infected + vehicle (*n* = 12), infected + α-aminoadipate (30 nmol, *n* = 6), infected + α-aminoadipate (100 nmol, *n* = 6), infected + minocycline (50 μg, *n* = 6), and infected + minocycline (150 μg, *n* = 6). The effects of most effective doses of glial inhibitors (100 nmol for α-aminoadipate and 150 μg for minocycline) were also tested in contralateral mechanical hyperalgesia and thermal hyperalgesia and paw edema (*n* = 24; *n* = 6 for non-infected group, *n* = 6 for infected vehicle-treated, *n* = 6 for α-aminoadipate-treated, and *n* = 6 for minocycline-treated). After defining the optimal dose of α-aminoadipate and minocycline to inhibit the hyperalgesia (100 nmol and 150 μg, respectively), and with an additional group treated with PDTC (NFκB inhibitor, 300 μg per mouse, i.t.), at the 30th day post-infection, the effects of these inhibitors on GFAP, Iba-1, TNF-α, IL-1β, CX_3_CL1 and CX_3_CR1 mRNA expression, and NFκB activation in spinal cord samples were evaluated (*n* = 60; *n* = 12 for non-infected group and *n* = 12 for infected vehicle-treated, α-aminoadipate-treated, minocycline-treated, and PDTC-treated groups). Finally, CX_3_CL1 mRNA expression in bilateral DRG cells of infected and control non-infected animals was evaluated at the 30th day post-infection (*n* = 18; *n* = 6 for non-infected group, *n* = 6 for infected ipsilateral, and *n* = 6 for infected contralateral). One last round of experiment evaluated whether the i.t. injection of TNF-α and IL-1β induces CX_3_CL1 mRNA expression in DRG (3 h after) and spinal cord (3–24 h after). Samples of TNF-α-stimulated (1 ng, i.t., *n* = 18) and IL-1β-stimulated (1 ng, i.t., *n* = 18) were compared to naïve non-infected animals (naïve control [*n* = 6] and vehicle [*n* = 18]). Samples of spinal cord used in the present study were from the L_4_–L_6_ segment, responsible for paw innervation. The experimental design, times of behavioral analysis, paw edema measurement, sample collection, and doses described above were based on previous studies [2, 16]. Each mouse was considered a unit. Every experiment was performed twice to verify reproducibility, and the informed *n* indicates the number of mice per group in each experiment. Experimenters were blinded to the treatments.

### Electronic pressure meter test for mechanical hyperalgesia

The mechanical hyperalgesia test consisted of evoking in mice placed in acrylic cages (12 × 10 × 17 cm^3^) with wire grid floor a hind paw flexion reflex with a handheld force transducer adapted with 0.5 mm^2^ polypropylene tip (electronic anaesthesiometer; Insight, Ribeirão Preto, SP, Brazil) [26]. The endpoint is characterized by the removal of the paw followed by clear flinching movements. The intensity of the pressure was automatically recorded after the paw withdrawal, and the value for the response was obtained by averaging three measurements. Mice were tested before and after treatments. In the first set of experiments, mechanical hyperalgesia was evaluated before and during 40 days after experimental infection, and subsequently, in the next phase, it was evaluated only at day 30 before and after (1–7 h) i.t. treatment with vehicle, α-aminoadipate, or minocycline. Results are expressed as δ (Δ) withdrawal threshold (in grams) calculated by subtracting the mean measurements at indicated time points from the basal mean measurements [2]. Mean withdrawal threshold between control non-infected and infected groups analyzed was 9.1 ± 0.5 g (mean ± SEM; 10 groups; *n* = 60) before inoculation of *L. amazonensis*. The experimenter was blinded to the treatments.

### Evaluation of thermal hyperalgesia

Thermal hyperalgesia was evaluated in mice as described previously [27]. Thermal hyperalgesia evaluation protocol was initially applied before and during 40 days after experimental infection, and subsequently, in the nest phase, it was evaluated only at day 30, before and after (1–7 hours) i.t. treatment with vehicle, α-aminoadipate, or minocycline. Briefly, mice were placed in a hot plate apparatus (EFF 361, Insight, Ribeirão Preto, SP, Brazil) maintained at 55 ± 1 °C. The reaction time was registered when the animal presents the behaviors of licking or flinching the infected hind paw. A maximum latency (cutoff) was set at 15 s to avoid tissue damage.

### Paw edema assessment

The paw edema was measured in mice as described previously [27]. In the first set of experiments, paw edema was evaluated before and during 40 days after experimental infection, and subsequently, in the next phase, it was evaluated only at day 30, before and after (1–7 h) i.t treatment with vehicle, α-aminoadipate, or minocycline. The measurements were made using a caliper (Digmatic Caliper, Mitutoyo Corporation, Kanagawa, Japan). Paw thickness was expressed as the difference (Δ reaction) in millimeters (mm) between the values obtained before (basal) and after the experimental infection.

### Histopathology

**Fig. 1:**
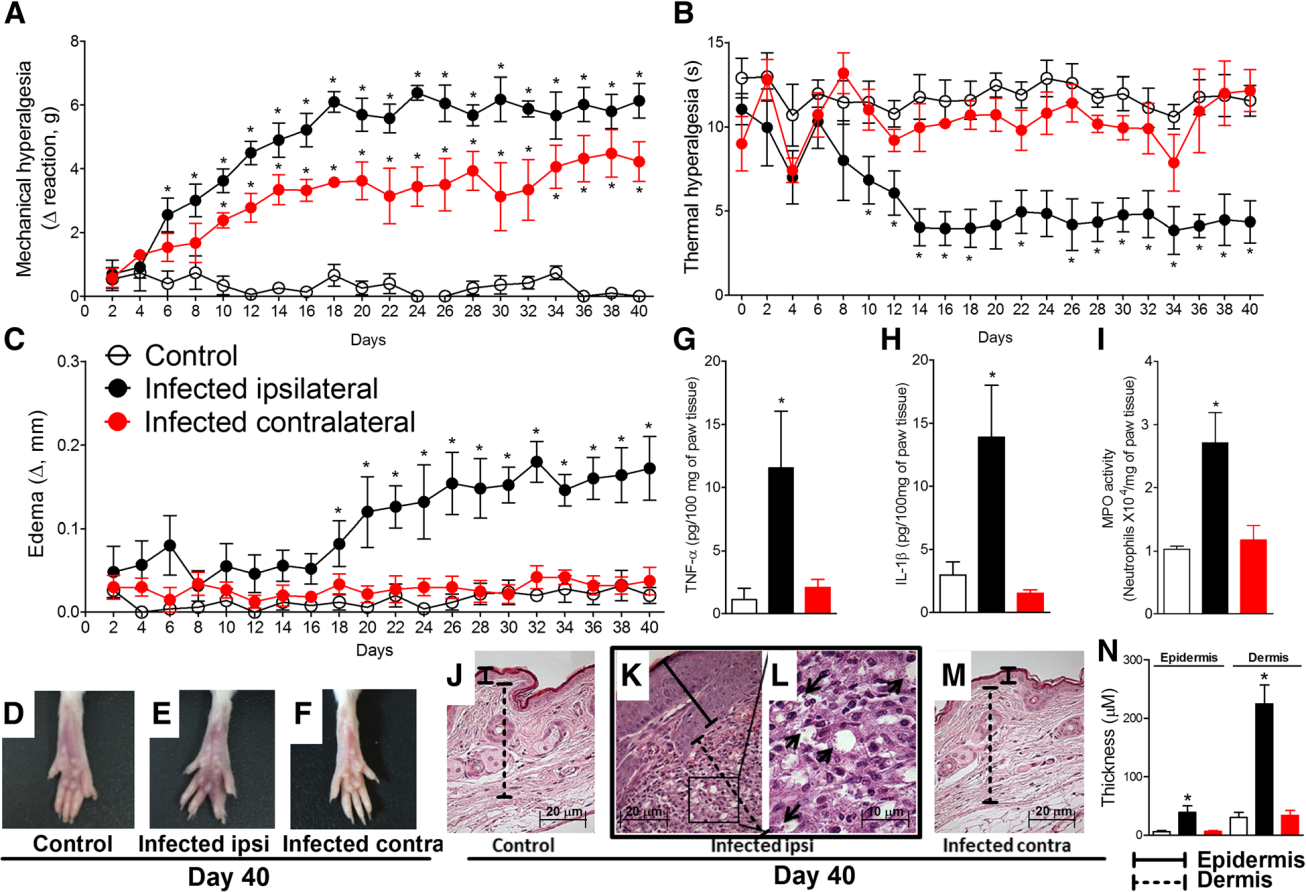
*L. amazonensis* induces hyperalgesia and paw inflammation in BALB/c mice. Mechanical hyperalgesia (**a**), thermal hyperalgesia (**b**), and paw edema (**c**) were measured bilaterally in control non-infected and infected mice during 40 days after the infection. Representative images of control non-infected mice (**d**) and ipsilateral (**e**) and contralateral sides (**f**) of infected mice paws as well as TNF-α (**g**), IL-1β (**h**), and MPO (**i**) levels in the paw tissue were evaluated bilaterally on day 40 post-infection by ELISA and colorimetric assays, respectively. Representative histological images (H&E) of control non-infected mice (**j**) and ipsilateral (**k** and **l**) and contralateral sides (**m**) of infected mice paw tissues are presented (four slides per mice/four mice per group). Magnification × 40 for **j**, **k**, and **m**, and × 100 for **l**. Continuous and dashed lines in **j**, **k**, and **m** delimit the extension of epidermis and dermis, respectively. Arrows in **l** indicate amastigote forms of *L. amazonensis* inside the vacuoles of macrophages. The quantification of epidermal and dermal thickening is presented as histopathological score in **n**. Results are presented as mean ± SEM of six mice per group per experiment and are representative of two separated experiments. *p < 0.05 compared to control non-infected mice (one-way ANOVA followed by Tukey post hoc)

Paw tissue samples were removed at the 40th day post-infection, fixed in 4% formaldehyde and processed for paraffin embedding. Tissue longitudinal sections (5 μm) were prepared in cryostat (CM1520, Leica Biosystem, Richmond, IL, USA) and slides stained with hematoxylin and eosin (H&E). The analysis of the slides (four slides per mice/four animal per group) was performed using light microscopy (Olympus Life Science, model CX31RTSF, Tokyo, Japan) with magnification of × 40 on the panels j, k, and m (scale bars 20 μm) and × 100 on the panel l (scale bar 10 μm) and presented in Fig. 1. Histopathological score of epidermis and dermis thickening was also performed for the experimental groups with magnification of × 200 and presented in panel n of Fig. 1.

### Cytokine measurement

Paw tissue samples were collected 40 days after the infection with *L. amazonensis*, homogenized in 500 μL of the appropriate buffer containing protease inhibitors and centrifuged (3000 rpm × 10 min × 4 °C). Resultant supernatants were used for the determination of TNF-α and IL-1β concentrations by enzyme-linked immunosorbent assay (ELISA) using paired antibodies as instructed by the manufacturer (eBioscience kits, Affymetrix, San Diego, CA, USA). For blood analysis, samples were collected by cardiac puncture 5–40 days after the infection and added into sterile microtubes containing anticoagulant (EDTA, 5000 IU/mL) for subsequent centrifugation for separation of plasma (500*g* × 20 min × 4 °C). In brief, 96-well plates were coated overnight at 4 °C with immunoaffinity-purified polyclonal sheep antibody especially for each cytokine evaluated. After blocking the plates, recombinant murine standards for each cytokine tested at various dilutions together with the samples were added in duplicate and incubated overnight at 4 °C. Rabbit biotinylated immunoaffinity-purified antibodies anti-TNF-α and anti-IL-1β were added followed by incubation at room temperature for 1 h. Then, 50 μL of avidin-HRP (1:5000 dilution) was added to each well; after 30 additional minutes, the plates were washed and the color reagent o-phenylenediamine (200 μL/well; Sigma-Aldrich, St. Louis, MO, USA) was added. After 5 min, the reactions were blocked with 1 M H_2_SO_4_ and measured spectrophotometrically (MultiSkan GO Microplate Spectrophotometer, ThermoScientific, Vantaa, Finland) at 450 nm. The results were expressed as picograms (pg) of cytokine per 100 mg of paw tissue and as picograms of cytokine per milliliter (mL) of plasma [28].

### MPO activity assay

The leukocyte migration to paw tissue was determined 40 days after *L. amazonensis* infection using the MPO kinetic-colorimetric assay following previous description [28]. Samples of paw tissue were collected in 50 mM K_2_HPO_4_ buffer (pH 6.0) containing 0.5% hexadecyl trimethylammonium bromide (HTAB) and kept at − 86 °C until the next step. Samples were then homogenized and centrifuged (16,100*g* × 2 min × 4 °C), and 10 μL of the resulting supernatant was mixed with 200 μL of 50 Mm phosphate buffer (pH 6.0), containing 0.167 mg/mL of O-dianisidine dihydrochloride and 0.0005% of hydrogen peroxide (H_2_O_2_) and assayed spectrophotometrically for MPO activity determination at 450 nm (MultiSkan GO Microplate Spectrophotometer, ThermoScientific, Vantaa, Finland). The MPO activity of paw tissue samples was compared to a standard curve of neutrophils, and the results were presented as MPO activity (number of neutrophils × 10^4^/mg of paw tissue).

### Reverse transcription and quantitative polymerase chain reaction

Reverse transcription and quantitative polymerase chain reaction (RT-qPCR) was performed following the protocol as described previously [2]. Spinal cord samples (L_4_–L_6_ entire segments) were collected initially 5–40 days and later only at day 30 after the infection with *L. amazonensis* for the determination of temporal profile of GFAP and Iba-1 mRNA expression and evaluation of CX_3_CL1, GFAP, Iba-1, TNF-α, and IL-1β mRNA expression, respectively. The purity of total RNA was measured with a spectrophotometer (MultiSkan GO Microplate Spectrophotometer, ThermoScientific, Vantaa, Finland), and the wavelength absorption ratio (260/280 nm) was between 1.8 and 2.0 for all preparations. Reverse transcription of total RNA to cDNA and qPCR were carried out using Go Taq® 2-Step RT-qPCR system (Promega Corporation, Madison, WI, USA) following the manufacturer’s instructions. The relative gene expression was measured using the comparative 2^−(ΔΔCq)^ method. The primers used are presented in Table 1. The expression of β-actin RNA was used as a reference gene to normalize data.

**Table 1:** Mouse mRNA primers used for RT-qPCR

Target gene	Forward	Reverse
β-actin	5′-AGCTGCGTTTTACACCCTTT-3′	5′-AAGCCATGCCAATGTTGTCT-3′
GFAP	5′-GCGCTCAATGCTGGCTTCA-3′	5′-TCTGCCTCCAGCCTCAGGTT-3′
Iba-1	5′-TGGAGTTTGATCTGAATGGAAAT-3′	5′-CAGGGCAGCTCGGAGATAGCTTT-3′
CX_3_CL1	5′-ATTGGAAGACCTTGCTTTGG-3′	5′-GCCTCGGAAGTTGAGAGAGA-3′
CX_3_CR1	5′-CACCATTAGTCTGGGCGTCT-3′	5′-GATGCGGAAGTAGCAAAAGC-3′
TNF-α	5′-TCTCATCAGTTCTATGGCCC-3′	5′-GGGAGTAGACAAGGTACAAC-3′
IL-1β	5′-GAAATGCCACCTTTTGACAGTG-3′	5′-TGGATGCTCTCATCAGGACAG-3′
ATF3	5′-CGAAGACTGGAGCAAAATGATG-3′	5′-CAGGTTAGCAAAATCCTCAAATAC-3′
JW11/JW12	5′-CCTATTTTACACCAACCCCCAGT-3′	5′-GGGTAGGGGCGTTCTGCGAAA-3′

### DNA extraction and parasite quantification by real-time qPCR

Real-time qPCR was conducted to evaluate the tissue parasite burden in ipsilateral draining lymph node, spleen, and contralateral lymph node starting from the day of the inoculation of the parasite until the 40th day post-infection. Organs were weighed, washed in PBS, and homogenized in lysis buffer (50 mM Tris-HCl [pH 7.6], 10 mM EDTA, 0.5% SDS, and 0.2 mg/mL proteinase K [Invitrogen, Carlsbad, CA]), followed by phenol-chloroform extraction of DNA. Samples were then homogenized and incubated at 55 °C for 12 h and subsequently extracted twice with phenol-chloroform-isoamyl alcohol (25:24:1). Two volumes of cold ethanol (Merck) were added to the aqueous phase, and samples were stored at − 20 °C for 12 h. Samples were then centrifuged for 30 min at 10,000*g*, washed with 70% ethanol, dried at room temperature, and resuspended in 10 mM Tris HCl (pH 8.5). Real-time qPCR was performed by using Platinum SYBR Green qPCR SuperMix UDG with ROX reagent (Invitrogen Corporation, New York, NY) with 100 ng total genomic DNA (gDNA). The quantification of parasites was carried out through the use of specific *Leishmania* primers described in Table 1. The samples were amplified with a Corbett Rotor-Gene thermal cycler under standardized steps. A standard curve constructed with DNA from culture samples of *L. amazonensis* promastigote forms was used for the determination of parasite load in evaluated tissues. Results were presented as parasites equivalents/100 nanograms (ng) of DNA per sample.

### CCI model

For CCI protocol, mice were anesthetized with ketamine and xylazine (10 μg/10 mL) followed by trichotomy in the surgery area. The incision was performed in the rear leg, and the sciatic nerve was exposed with a glass rod. A moderate constriction injury was performed around the sciatic nerve with a chrome suture according to the method described by Bennett and Xie [29] adapted to mice [16].

### Western blot assay

At the 30th day after the infection with *L. amazonensis*, L_4_–L_6_ entire segments of the spinal cord were dissected and the whole sample homogenized in RIPA buffer containing protease and phosphatase inhibitors. The lysates were then homogenized and centrifuged (0.5*g* for 10 min at 4 °C). The protein extracts were separated by SDS-PAGE and transferred onto a nitrocellulose membrane (GE Healthcare-Amersham, Pittsburgh, PA, USA). The membranes were then incubated in blocking buffer 95% non-fat milk in Tris-buffered saline with Tween 20 or 1% bovine serum albumin (BSA) for different times for each antibody at 4 °C in the presence of primary antibody. β-Actin, GFAP, and IκBα were purchased from Cell Signaling Technology (Danvers, MA, USA), and Iba-1 and secondary antibody (anti-rabbit, HRP conjugated) were purchased from Thermo Fisher Scientific (Waltham, MA, USA). Catalog numbers are indicated below. The antibodies and Western blot conditions were as follows: β-actin (#4970, 1:1000) on 12% gel and blocked with 5% non-fat milk; GFAP (#12389, 1:1000) on 12% gel and blocked with 5% non-fat milk; Iba-1 (#PA5-27436, 1:1000) on 15% gel and blocked with 5% non-fat milk; and total IκBα (#9242, 1:1000) on 10% gel and blocked with 5% BSA. The molecular masses of protein were confirmed by Precision Plus Protein Standards (Bio-Rad, Hercules, CA, USA). After washing in Tris-buffered saline (TBS) with Tween 20, the membrane was incubated with secondary antibody (anti-rabbit, #31460, 1:2000) for 2 h at room temperature. Protein was visualized by chemiluminescence with ECL detection reagent (Luminata™ Forte, Millipore, USA). The membranes were reprobed with antibody against β-actin for use as loading control in addition to loading the same amount of protein.

### Spinal cord immunofluorescence

On day 30 after the infection with *L. amazonensis*, mice were perfused through the ascending aorta with saline followed by 4% of paraformaldehyde. After the perfusion, L_4_–L_6_ segments of the spinal cord were dissected out and post-fixed and then replaced overnight with 30% sacarose. The spinal cord segments were embedded in optimum cutting temperature (O.C.T.) using Tissue-Tek® compound (Sakura® Finetek USA, Torrance, CA), and 7-μm sections were cut in a cryostat (CM1520, Leica Biosystem, Richmond, IL, USA) and processed for immunofluorescence (four slides per mice/four animal per group). All of the sections were blocked with a buffer solution (500 μL per slide containing PBS plus 0.1% Tween 20 plus 5% BSA) for 2 h at room temperature and subsequently incubated overnight at − 4 °C with a solution containing primary antibodies against GFAP (#180063; 1:500 dilution; Invitrogen, Life Technologies, Carlsbad, CA, USA) and Iba-1 (#PA5-27436; 1:500 dilution, Invitrogen, Life Technologies, Carlsbad, CA, USA). Next, a new incubation with secondary antibody (Alexa Fluor 488, #A-110088 1:1000 dilutions, Thermo Fisher Scientific, Waltham, MA, USA) was performed for 1.5 h at room temperature. The slide assembly was carried out using ProLong^TM^ Gold Antifade Mountant with DAPI melting media (#P36931, Thermo Fisher Scientific, Waltham, MA, USA). Immunofluorescence analyses were performed in the dorsal horn of the spinal cord. Dashed lines were used in some representative images for the demonstration of the gray and white matter areas in the dorsal horn of the spinal cord in which leishmaniasis-induced glial cell activation was observed and analyzed. For quantification of GFAP and Iba-1 immunostainings in Figs. 3 and 4, equivalent areas of the spinal cord in control non-infected and infected mice were selected. For Figs. 3 and 4, magnification of × 10 on the panels d and e (scale bars 250 μm) and × 20 on the panels f–k (scale bars 100 μm with gradual additional zoom on the panels h–k). The images and analysis were performed using a confocal microscope (SP8, Leica Microsystems, Mannheim, Germany) [16].

### NFκB activation assay

The determination of NFκB activation in the spinal cord samples was performed following the protocol as described previously [2]. Spinal cord samples (L_4_–L_6_ entire segments) were collected at day 30 after the infection with *L. amazonensis* and the whole sample homogenized in ice-cold lysis buffer (Cell Signaling Technology, Beverly, MA, USA). The homogenates were centrifuged (16,000*g* × 10 min × 4 °C) and the supernatants use to assess the levels of total and phosphorylated NFκB p65 subunit by ELISA using PathScan kits (Cell Signaling Technology, Beverly, MA, USA) according to the manufacturer’s instructions. The results were expressed as total-p65/phosphor-p65 ratio measured spectrophotometrically (MultiSkan GO Microplate Spectrophotometer, ThermoScientific, Vantaa, Finland) at 450 nm.

### Statistical analysis

Results are presented as means ± SEM of measurements made on three to six mice in each group depending on the analysis, per experiment, and are representative of two separate experiments. Two-way analysis of variance (ANOVA) was used to compare the groups and doses at all times when responses were measured at different times after the parasite injection. Analyzed factors were treatments, time, and time versus treatment interaction, and when interaction was significant, one-way ANOVA followed by Tukey’s post hoc was performed for each time point. Differences between responses were evaluated by one-way ANOVA followed by Tukey’s post hoc for data of single time point. Statistical differences were considered significant when *P* < 0.05.

## Results

### *L. amazonensis* i.pl. infection induces chronic mechanical and thermal hyperalgesia associated to the development of paw edema and increases TNF-α, IL-1β, and MPO activity levels in the paw tissue at the 40th day post-infection

The measurements of mechanical hyperalgesia, thermal hyperalgesia, and paw edema were performed between 2 and 40 days after i.pl. infection with *L. amazonensis* (Fig. 1a–f). Samples of paw tissue were collected at day 40 after the infection to evaluate TNF-α and IL-1β production, MPO activity levels, and histopathological analysis (Fig. 1g–m). *L. amazonensis* infection induced chronic ipsilateral mechanical and thermal hyperalgesia compared to control non-infected animals starting at days 6 and 10, respectively, and persisting until day 40 post-infection (*p* < 0.05, Fig. 1a, b). Interestingly, chronic mechanical hyperalgesia but not thermal hyperalgesia (*p* < 0.05, Fig. 1a, b) was detected in the contralateral paw of the infected animals compared to control non-infected animals from the 10th to 40th days post-infection. These results suggest that pain induced by leishmaniasis could involve the activation of the ipsilateral spinal cord side to the infection and also a minor activation of the contralateral spinal cord side to the infection. The paw edema became evident from day 18 onwards, remaining significantly different from the control non-infected animals until the 40th day post-infection (*p* < 0.05, Fig. 1c). Paw edema was not observed in the contralateral paw of infected animals (Fig. 1c). Representative pictures from control non-infected animals, swollen ipsilateral paw, and contralateral paw of infected animals at day 40 post-infection are presented respectively in Fig. 1d–f. *L. amazonensis* infection also increased the levels of TNF-α and IL-1β, and MPO activity in the ipsilateral, but not contralateral paw tissue of infected mice when compared to control non-infected animals at day 40 after the infection (*p* < 0.05, Fig. 1g–i). Histopathological images of ipsilateral paw tissue of infected animals (Fig. 1k, l) show the presence of amastigote forms of *L. amazonensis* inside the vacuoles of macrophages (arrows). Paw tissue of control non-infected (Fig. 1j) and contralateral paw of infected animal (Fig. 1m) presented regular histology. Histopathological score presented in Fig. 1n shows increased thickening of the epidermis and dermis in ipsilateral paw compared to control non-infected and contralateral paw of infected animals. In addition to supporting the hyperalgesic and edematogenic effects of *L. amazonensis* infection [2], these data evidenced increases in TNF-α and IL-1β production in parallel to increased neutrophil recruitment (as reflected by MPO assay) in infected paw tissue. Therefore, although leishmaniasis induces chronic ulcerative infection, these results confirm that there is consistent peripheral infection-induced inflammation even at early time points when skin ulceration is absent.

### *L. amazonensis* i.pl. infection does not change the mRNA expression of ATF3 in the DRG

The extent that DRGs were affected by *L. amazonensis* i.pl. infection was evaluated 30 days of infection [2]. There was no significant induction of ATF3 mRNA expression in DRG samples of infected mice compared to non-infected mice (Additional file 1: Figure S1). CCI-induced ATF3 mRNA expression in the DRG was used as a positive control of ATF3 mRNA expression (Additional file 1: Figure S1). The absence of increased mRNA expression of ATF3 in ipsilateral DRG suggests that at least during this period of the disease course (30th day), there is no nerve damage, since this transcription factor is expressed by injured neurons [30].

### *L. amazonensis* i.pl. infection does not induce increases in plasmatic TNF-α and IL-1β levels and induced a time-dependent increase in parasite load in the ipsilateral draining lymph node, without significant time-dependent increases in the spleen and contralateral lymph node

The blood of infected animals was collected 5–40 days after the infection for the evaluation of plasmatic levels of TNF-α and IL-1β over the course of the disease (Additional file 2: Figure S2). The i.pl. infection with *L. amazonensis* does not modify the blood levels of TNF-α (Additional file 2: Figure S2A) and IL-1β (Additional file 2: Figure S2B) during the experimental protocol in comparison with non-infected animals, suggesting that the systemic levels of these hyperalgesic cytokines have no decisive role in the development of neuroinflammation in the spinal cord in the present model. Regarding parasitism, we investigated parasite load at different time points (0, 5, 10, 20, 30, and 40 days) post-infection in immune organs distant from the site of inoculation of the parasite (ipsilateral draining lymph node, spleen, and contralateral lymph node) to analyze the occurrence of systemic infection (Additional file 3: Figure S3). Mice received i.pl. injection of parasites on day 0. The presence of parasites was not detected in samples collected at the day of infection (Additional file 3: Figure S3A–C). The presence of parasites occurred in all evaluated organs at the fifth day post-infection onwards. However, the parasitism remained stable except by the ipsilateral draining lymph node that presented a progressive and time-dependent increase of parasitism (Additional file 3: Figure S3A–C). These results might be related to the time-dependent increase of infection in the paw [2].

### The i.t. treatments with anti-CX_3_CL1 antibody, etanercept, and IL-1ra inhibited *L. amazonensis*-induced ongoing mechanical and thermal hyperalgesia without affecting paw edema

**Fig. 2:**
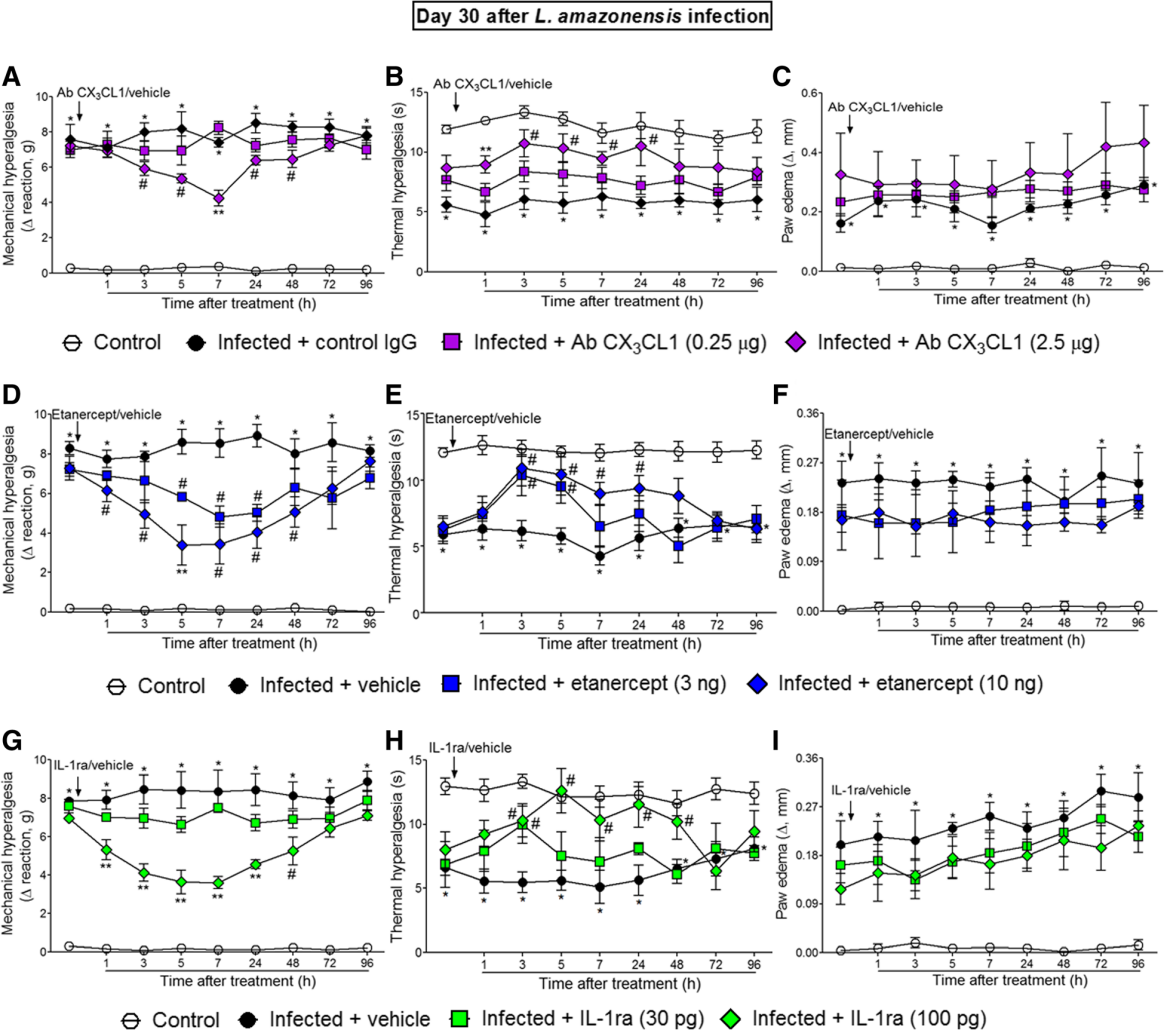
Neutralizing antibody anti-CX_3_CL1, etanercept, and IL-1ra i.t. treatments inhibit *L. amazonensis*-induced hyperalgesia, but not paw edema. Mechanical (**a**, **d**, and **g**) and thermal (**b**, **e**, and **h**) hyperalgesia and paw edema (**c**, **f**, and **i**) were measured in control non-infected and infected mice at day 30 after the infection, and subsequently, infected mice received i.t. injection of antibody anti- CX_3_CL1 (0.25–2.5 μg), etanercept (3–10 ng), IL-1ra (30–100 pg), or vehicles (control IgG for Ab CX_3_CL1 and saline for the other drugs) followed by measurement of mechanical and thermal hyperalgesia and paw edema. Results are presented as mean ± SEM of six mice per group per experiment and are representative of two separate experiments. *p < 0.05 compared to control non-infected mice; ^#^*p* < 0.05 compared to vehicle-treated infected mice; **p < 0.05 compared to the lower doses of antibody anti- CX_3_CL1, etanercept, IL-1ra, and vehicle-treated infected mice (one-way ANOVA followed by Tukey post hoc)

Mice were treated by i.t. route with vehicles (control IgG or saline) or anti-CX_3_CL1 antibody, etanercept, and IL-1ra at day 30 after *L. amazonensis* infection, and mechanical hyperalgesia, thermal hyperalgesia, and paw edema were evaluated (Fig. 2). This time point was selected because it is when TNF-α expression in the spinal cord reaches its peak [2]. The dose of 0.25 μg of anti-CX_3_CL1 antibody had no effect on mechanical hyperalgesia and thermal hyperalgesia, while the dose of 2.5 μg significantly inhibited mechanical hyperalgesia and thermal hyperalgesia between 3–48 and 1–24 h after the treatment, respectively. The anti-hyperalgesic effect of the 2.5 μg of anti-CX_3_CL1 antibody was also significant compared to the 0.25 μg dose at the seventh and at the first hours after the treatment for mechanical hyperalgesia and thermal hyperalgesia, respectively (*p* < 0.05, Fig. 2a, b). Antibody (IgG) control treatment presents no effect. The dose of 3 ng of etanercept inhibited mechanical hyperalgesia between 5 and 24 h and thermal hyperalgesia between 3 and 5 h after the treatment. On the other hand, significant inhibition of mechanical hyperalgesia and thermal hyperalgesia occurred for a longer duration with the dose of 10 ng of etanercept, which was observed between 1–48 and 3–24 h after the treatment, respectively, in comparison with the vehicle (saline)-treated group (*p* < 0.05, Fig. 2d, e). The effect of 10 ng of etanercept was also significant compared to the lower dose at the fifth hour after the treatment for mechanical hyperalgesia (*p* < 0.05, Fig. 2d). The dose of 30 pg of IL-1ra had no effect on mechanical hyperalgesia, but inhibited thermal hyperalgesia at the third hour after the treatment, whereas the dose of 100 pg inhibited mechanical hyperalgesia and thermal hyperalgesia between 1–48 and 3–48 h after the treatment, respectively, in comparison with the vehicle (saline)-treated group (*p* < 0.05, Fig. 2g, h). The dose of 100 ng of IL-1ra also reduced mechanical hyperalgesia compared to the dose of 30 ng between 1 and 24 h after the treatment (*p* < 0.05, Fig. 2g). None of the treatments affected *L. amazonensis*-induced paw edema compared to vehicle-treated groups (Fig. 2c, f, and i). These results suggest the participation of spinal cord CX_3_CL1, TNF-α, and IL-1β in the mechanism of *L. amazonensis*-induced pain.

### *L. amazonensis* i.pl. infection induces the activation of astrocytes in the spinal cord

**Fig. 3:**
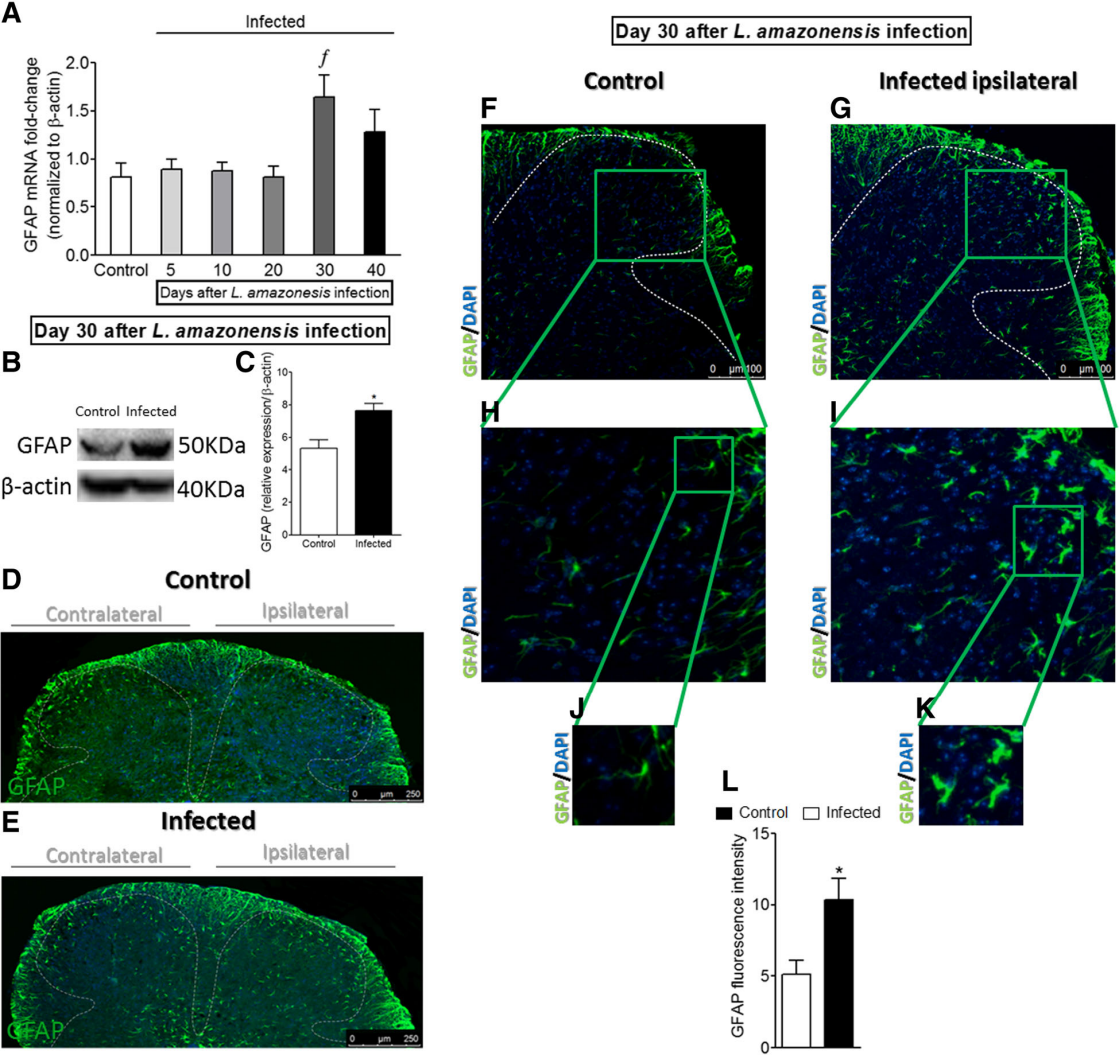
Detection of spinal cord GFAP induced by i.pl. *L. amazonensis* infection. GFAP mRNA expression was determined in control non-infected and infected mice after the infection (5–40 days) by RT-qPCR (**a**). On day 30 after the infection (peak of GFAP mRNA expression), Western blot analysis of the whole spinal cord was conducted to confirm GFAP expression at day 30 post-infection (three mice per group, **b** and **c**). Subsequently, spinal cord samples were stained with primary antibody for astrocytes (GFAP, green) and regular nucleus (DAPI, blue) detection (four mice per group/four slides per mice). Representative immunostainings of the spinal cord of control non-infected and infected mice are shown in **d**–**k** (**d** and **e** magnification × 10, scale bar 250 μm; panels **f**–**k** magnification × 20, scale bar 100 μm, with gradual zoom for panels **h** and **i** and **j** and **k**). **l** The percentage of GFAP fluorescent intensity in each experimental group. Results are presented as mean ± SEM of six mice per group per experiment and are representative of two separated experiments for **a**. For **b** and **c**, results are presented as mean ± SEM of three mice per group and are representative of two separated experiments. For **f**–**l**, results are presented as mean ± SEM of four mice per group and are representative of two separated experiments. ƒ*p* < 0.05 compared to control non-infected, and 5, 10, and 20 days infected groups; *p < 0.05 compared to control non-infected mice (one-way ANOVA followed by Tukey post hoc)

Whether *L. amazonensis* i.pl. infection induces the activation of spinal cord astrocytes is unknown. In this sense, the temporal profile of spinal cord GFAP mRNA expression over the course of disease (5–40 days post-infection) was investigated (Fig. 3a). RT-qPCR data showed no changes during the first 20 days when compared to control non-infected animals. However, significant increases in GFAP mRNA expression were detected at day 30 after the infection when compared to previous days (*p* < 0.05, 5–20) and control non-infected animals, followed by a slight reduction at day 40, without significant difference compared to other groups in this last day of evaluation (Fig. 3a). To confirm RT-qPCR data regarding astrocytes activation (GFAP, Fig. 3a) at day 30 after *L. amazonensis* infection, Western blot (Fig. 3b, c) and immunofluorescence (Fig. 3d–l) assays were performed in spinal cord samples of control non-infected and infected animals. Western blot analysis of the spinal cord tissues showed astrocyte activation in infected animals that was not detected in control non-infected animals (*p* < 0.05, GFAP, Fig. 3b, c). Immunofluorescence of the spinal cord evidenced that the activation of astrocytes (GFAP) occurred bilaterally in infected animals, which was not observed in control non-infected animals (Fig. 3d, e). Infected mice presented an increase of GFAP intensity of fluorescence in both gray and white matter areas of the ipsilateral side of the infection in the spinal cord (Fig. 3g, i, k, and l) compared to non-infected mice (Fig. 3f, h, j, and l). Together with the upregulation of GFAP expression, astrocyte activation or astrogliosis is characterized by cellular hypertrophy observed as enlarged cell bodies and thickening of process [31], and this was also observed in spinal cord samples of infected mice (*p* < 0.05, Fig. 3i, k). *L. amazonensis* infection induces spinal cord activation of astrocytes (Fig. 3k) not observed in control non-infected animals (Fig. 3j).

### *L. amazonensis* i.pl. infection induces the activation of microglia in the spinal cord

**Fig. 4:**
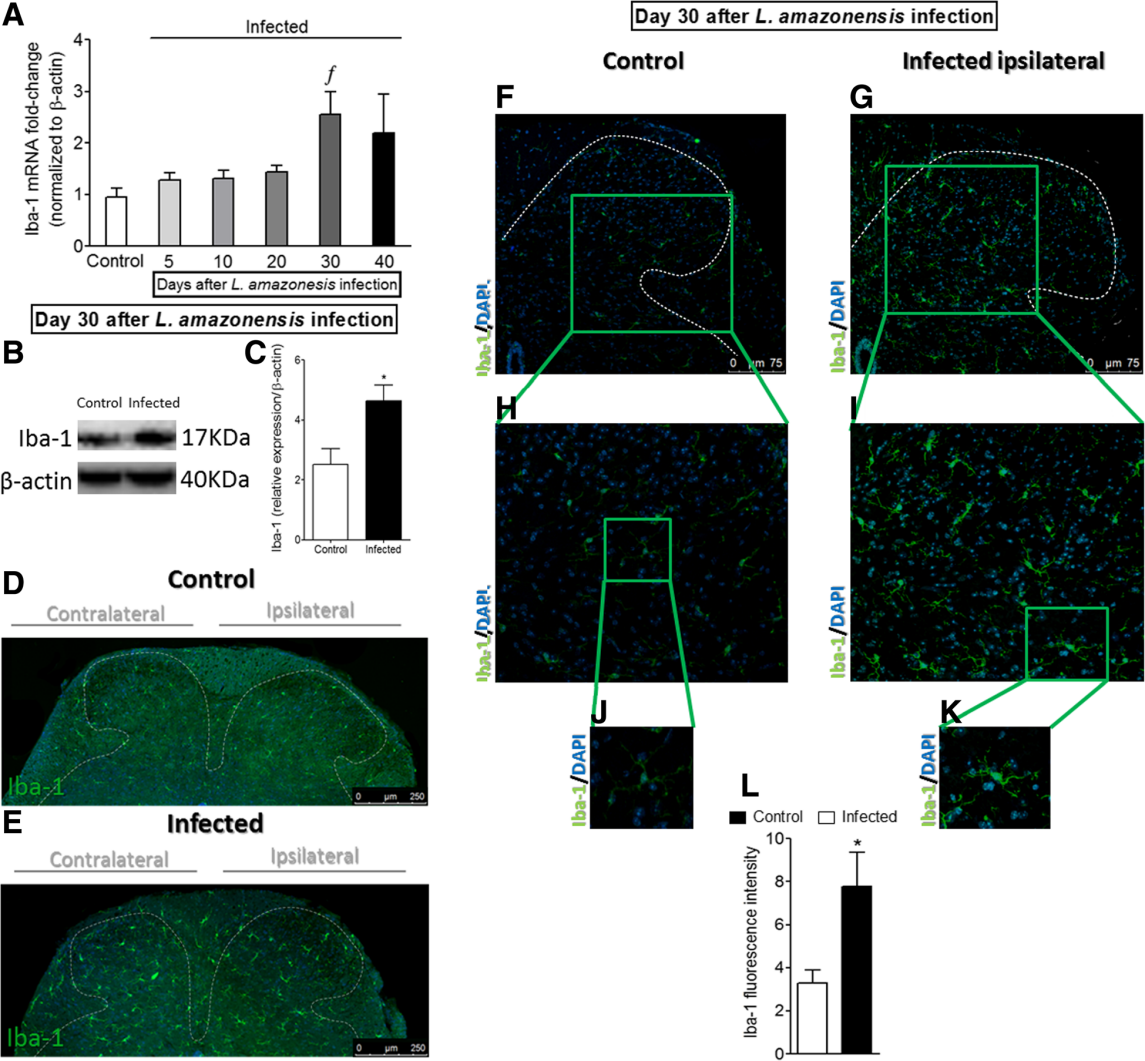
Detection of spinal cord Iba-1 induced by i.pl. *L. amazonensis* infection. Iba-1 mRNA expression was determined in control non-infected and infected mice after the infection (5–40 days) by RT-qPCR (**a**). On day 30 after the infection (peak of Iba-1 mRNA expression), Western blot analysis of the whole spinal cord was conducted to confirm Iba-1 expression at day 30 post-infection (four mice per group, **b** and **c**). Subsequently, spinal cord samples were stained with primary antibody for microglia (Iba-1, green) and regular nucleus (DAPI, blue) detection (four mice per group/four slides per mice). Representative immunostainings of the spinal cord of control non-infected and infected mice are shown in **d**–**k** (**d** and **e**, magnification × 10, scale bar 250 μm; panels **f**–**k** magnification × 20, scale bar 100 μm, with gradual zoom for panels **h** and **i** and **j** and **k**). **l** The percentage of Iba-1 fluorescent intensity in each experimental group. Results are presented as mean ± SEM of six mice per group per experiment and are representative of two separated experiments for **a**. For **b** and **c**, results are presented as mean ± SEM of four mice per group and are representative of two separated experiments. For panels **f**–**l**, results are presented as mean ± SEM of four mice per group and are representative of two separated experiments. ƒ*p* < 0.05 compared to control non-infected, and 5, 10 and 20 days infected groups; *p < 0.05 compared to control non-infected mice (one-way ANOVA followed by Tukey post hoc)

Following the same sequence of experiments as presented in the previous subsection, mice received i.pl. inoculation of *L. amazonensis* to evaluate whether the infection induces the activation of spinal cord microglia (Fig. 4 and Additional file 4: Figure S4). The temporal profile of Iba-1 mRNA expression during the course of disease was similar to that observed for GFAP. There was no statistical difference in the Iba-1 mRNA expression between 5 and 20 days after the infection when compared to the control group, while significant increase was observed at day 30 post-infection (*p* < 0.05). In the following time point (40th day), Iba-1 mRNA expression decreased slightly and presented no statistical difference compared to other groups (Fig. 4a). Spinal cord Western blot (Fig. 4b, c) and immunofluorescence (Fig. 4d–l) analysis at day 30 after *L. amazonensis* infection confirmed RT-qPCR data and demonstrated microglial activation in the infected group in comparison with control non-infected animals (*p* < 0.05, Iba-1, Fig. 4b–l), but contrary to what was evidenced for astrocytes (GFAP, Fig. 3g), microglial activation was detected almost exclusively in the gray matter area of the ipsilateral side of the infection in the spinal cord. Interestingly, as observed for astrocytes (GFAP, Fig. 3d, e), immunofluorescence of the spinal cord demonstrated bilateral microglial activation (Iba-1) in infected animals and not in control non-infected animals (Fig. 4d, e). Microglia activation is often reported as Iba-1 upregulation associated to profound changes in cell shape, reflected by hypertrophy of the cell body and increase of the branching processes [32], which can be observed in Fig. 4i and k (*p* < 0.05). Therefore, *L. amazonensis* infection induces spinal cord activation of microglia (Fig. 4j–l). CX_3_CR1 mRNA expression did not present statistical difference compared to the control group until the 10th day; however, a significant increase was observed between 20 and 30 days. At the 40th day, no statistical difference was observed between the groups (Additional file 1: Figure S4). CX_3_CR1 contributes to microglia activation [15]; thus, the increase of CX_3_CR1 mRNA expression before Iba-1 mRNA expression rise is reasonable.

### The i.t. treatment with the selective astrocyte inhibitor α-aminoadipate diminishes *L. amazonensis*-induced mechanical and thermal hyperalgesia without affecting paw edema

**Fig. 5:**
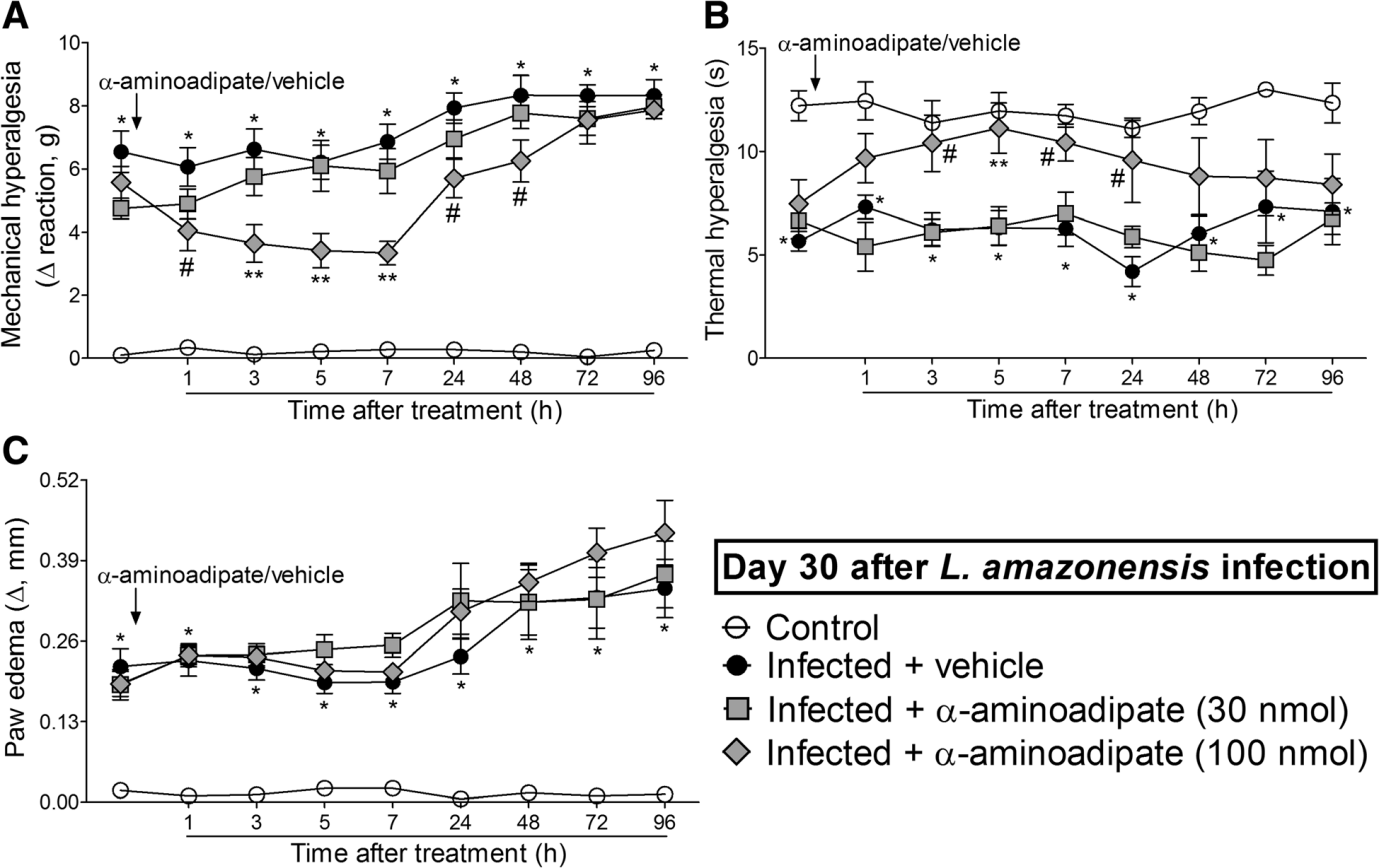
α-Aminoadipate i.t. treatment inhibits *L. amazonensis*-induced hyperalgesia, but not paw edema. Mechanical (**a**) and thermal (**b**) hyperalgesia and paw edema (**c**) were measured in control non-infected and infected mice on day 30 after the infection, and subsequently, infected mice received i.t. injection of α-aminoadipate (selective astrocyte inhibitor, 30–100 nmol) or vehicle for measurement of mechanical and thermal hyperalgesia and paw edema. Results are presented as mean ± SEM of six mice per group per experiment and are representative of two separate experiments. *p < 0.05 compared to control non-infected mice; ^#^*p* < 0.05 compared to vehicle treated-infected mice; **p < 0.05 compared to 30 nmol α-aminoadipate and vehicle-treated infected mice (one-way ANOVA followed by Tukey post hoc)

Mice were treated by i.t. route with vehicle or α-aminoadipate (30–100 nmol) 30 days after *L. amazonensis* infection, and mechanical hyperalgesia, thermal hyperalgesia, and paw edema were evaluated (Fig. 5). The dose of 30 nmol of α-aminoadipate did not affect mechanical hyperalgesia, thermal hyperalgesia, and paw edema (Fig. 5a–c). On the other hand, i.t. treatment with 100 nmol of α-aminoadipate significantly inhibited *L. amazonensis*-induced mechanical hyperalgesia for up to 48 h after the treatment and thermal hyperalgesia between 3 and 24 h after the treatment in comparison with the vehicle-treated group (*p* < 0.05, Fig. 5a, b). The anti-hyperalgesic effect of 100 nmol of α-aminoadipate in thermal hyperalgesia was also significant compared to the dose of 30 nmol at 5 h (*p* < 0.05, Fig. 5b). The treatment with α-aminoadipate did not affect *L. amazonensis*-induced paw edema (Fig. 5c). These results demonstrate that targeting spinal cord astrocytes inhibits *L. amazonensis*-induced hyperalgesia.

### The i.t. treatment with the microglia inhibitor minocycline diminishes *L. amazonensis*-induced mechanical and thermal hyperalgesia without affecting paw edema

**Fig. 6:**
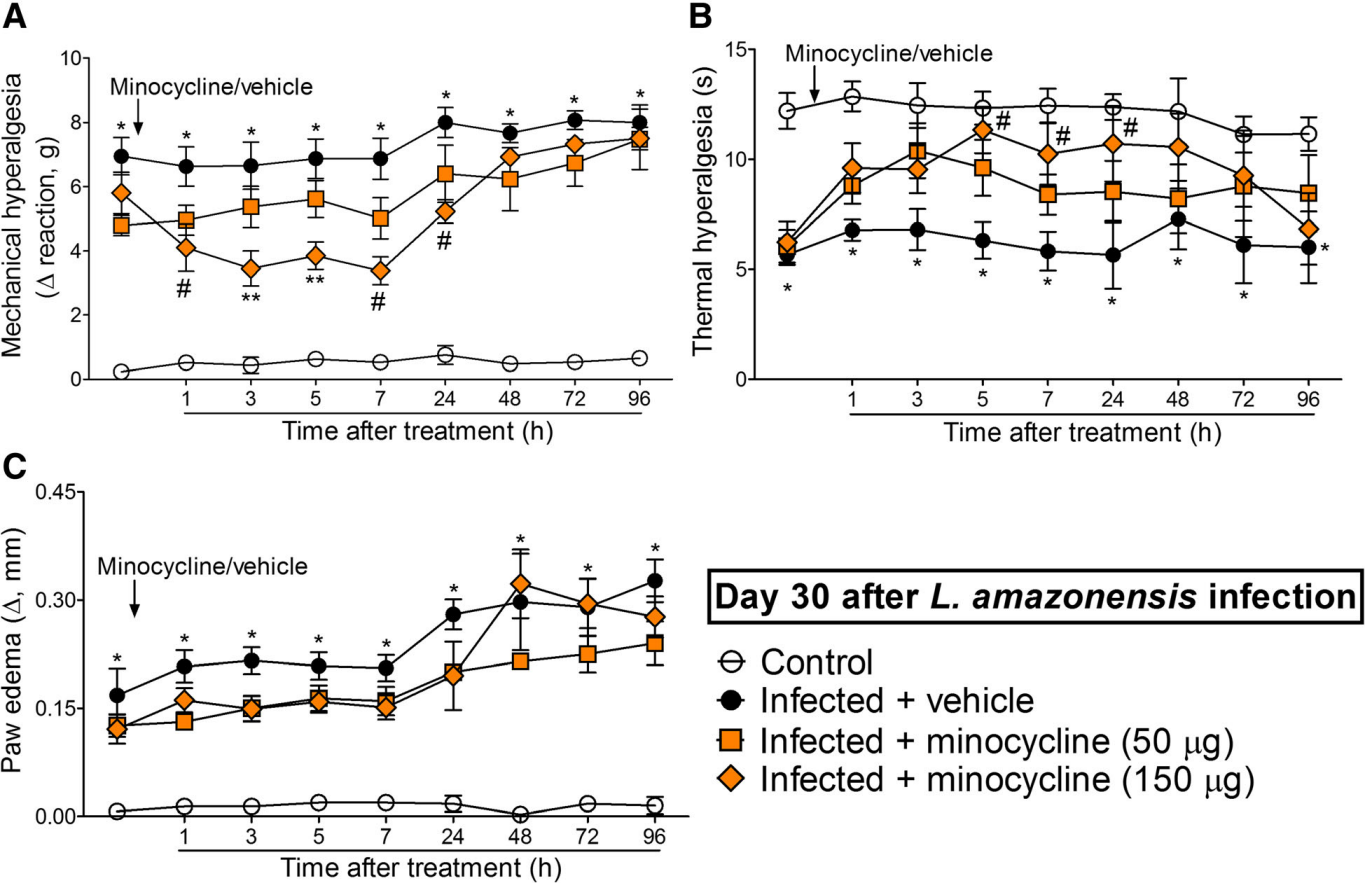
Minocycline i.t. treatment inhibits *L. amazonensis*-induced hyperalgesia, but not paw edema. Mechanical (**a**) and thermal (**b**) hyperalgesia and paw edema (**c**) were measured in control non-infected and infected mice on day 30 after the infection, and subsequently, infected mice received i.t. injection of minocycline (microglia inhibitor, 50–150 μg) or vehicle for measurement of mechanical and thermal hyperalgesia and paw edema. Results are presented as mean ± SEM of six mice per group per experiment and are representative of two separate experiments. *p < 0.05 compared to control non-infected mice; ^#^*p* < 0.05 compared to vehicle-treated infected mice; **p < 0.05 compared to 50 μg minocycline and vehicle-treated infected mice (one-way ANOVA followed by Tukey post hoc)

Mice were treated by i.t. route with vehicle or minocycline (50–150 μg) at the 30th day after *L. amazonensis* infection to determine the participation of spinal cord microglia in the mechanical hyperalgesia, thermal hyperalgesia, and paw edema in this infection (Fig. 6). The dose of 50 μg of minocycline did not alter leishmaniasis-induced mechanical and thermal hyperalgesia (Fig. 6a, b). On the other hand, i.t. treatment with 150 μg of minocycline inhibited *L. amazonensis*-induced mechanical and thermal hyperalgesia between 1–24 h and 5–24 h, respectively (*p* < 0.05, Fig. 6a, b). There was also significant inhibition of mechanical hyperalgesia comparing the doses of 50 and 150 μg of minocycline at the third and fifth hour after treatment (*p* < 0.05, Fig. 6a). Minocycline treatment did not alter *L. amazonensis*-induced paw edema (Fig. 6c). Therefore, inhibiting spinal cord microglial activity diminished *L. amazonensis*-induced hyperalgesia.

### The i.t. treatment with the selected effective analgesic doses of α-aminoadipate and minocycline diminishes *L. amazonensis*-induced contralateral mechanical hyperalgesia

The two previous experiments evaluating the dose-response effects of α-aminoadipate and minocycline upon ipsilateral hyperalgesia and paw edema showed that the higher doses tested for both drugs presented a satisfactory analgesic effect on *L. amazonensis*-induced hyperalgesia. Thus, we tested the effects of these same doses (150 nmol and 150 μg, respectively) upon the contralateral mechanical hyperalgesia in infected mice 7 h after the treatments (Additional file 5: Figure S5). Both α-aminoadipate and minocycline inhibited contralateral mechanical hyperalgesia from 3 to 48 h after the treatment at the 30th day post-infection (Additional file 5: Figure S5A). As expected, treatments with these glial inhibitors had no effect on contralateral thermal hyperalgesia and paw edema (Additional file 5: Figure S5B and C), since these parameters were not altered previously in the infected animals in the contralateral side. These data corroborate that activated spinal cord astrocytes and microglia (Figs. 3 and 4) mediate the mechanical hyperalgesia observed in the contralateral paw.

### Targeting spinal cord astrocytes, microglia, and NFκB inhibits *L. amazonensis*-induced spinal cord GFAP, Iba-1, TNF-α, IL-1β, CX_3_CR1, and CX_3_CL1 mRNA expression

**Fig. 7:**
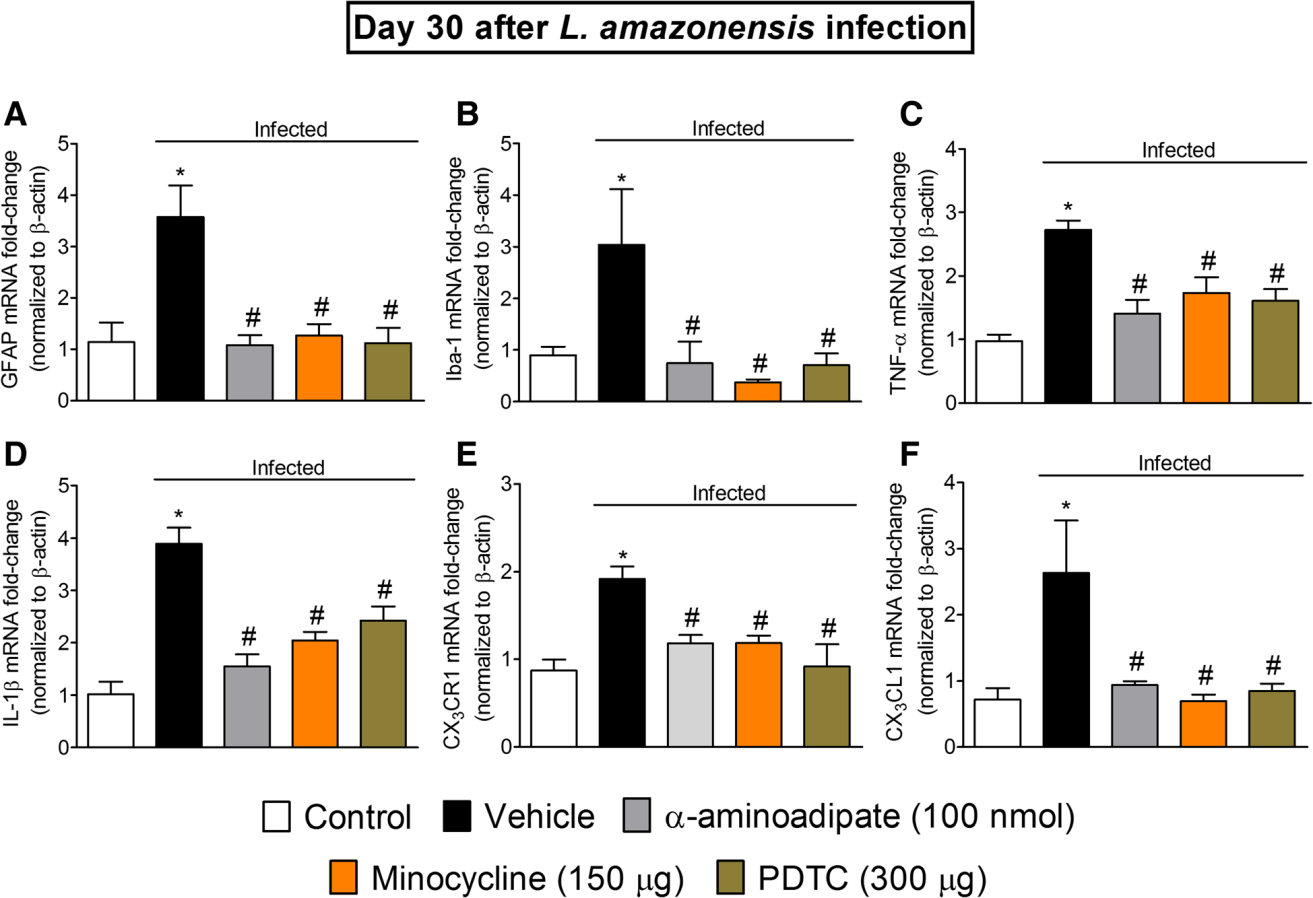
α-Aminoadipate, minocycline, and PDTC i.t. treatments inhibit *L. amazonensis*-induced spinal cord GFAP, Iba-1, TNF-α, IL-1β, CX_3_CR1, and CX_3_CL1 increased mRNA expression. On day 30 after the infection, spinal cord GFAP (**a**), Iba-1 (**b**), TNF-α (**c**), IL-1β (**d**), CX_3_CR1 (**e**), and CX_3_CL1 (**f**) mRNA expression were determined in control non-infected and infected mice 7 h after treatments with vehicle, α-aminoadipate, minocycline, or PDTC by RT-qPCR. Results are presented as mean ± SEM of six mice per group per experiment and are representative of two separate experiments. *p < 0.05 compared to control non-infected mice; ^#^*p* < 0.05 compared to vehicle-treated infected mice (one-way ANOVA followed by Tukey post hoc)

Considering the results obtained in dose-response experiments for α-aminoadipate and minocycline presented earlier, the doses of 100 nmol and 150 μg, respectively, were selected for the experiments in this section. The dose of the NFκB inhibitor PDTC of 300 μg per mouse by i.t. route was selected in a dose-response curve from a previous study [2]. Mice received i.t. treatment with α-aminoadipate, minocycline, or PDTC at the 30th day after *L. amazonensis* infection, and samples of the spinal cord (L_4_–L_6_) were collected 7 h after the treatments for RT-qPCR analysis of GFAP (Fig. 7a), Iba-1 (Fig. 7b), TNF-α (Fig. 7c), IL-1β (Fig. 7d), CX_3_CR1 (Fig. 7e), and CX_3_CL1 (Fig. 7f) mRNA expression. Leishmaniasis induced significant increase of spinal cord GFAP, Iba-1, TNF-α, IL-1β, CX_3_CR1, and CX_3_CL1 mRNA expression, which were inhibited by α-aminoadipate, minocycline, and PDTC treatments (*p* < 0.05, Fig. 7a–e). These results indicate that spinal cord astrocytes, microglia, and NFκB activation underlies the mRNA expression of pro-hyperalgesic cytokines and chemokines in leishmaniasis.

### Leishmaniasis induces CX_3_CL1 mRNA expression in the ipsilateral DRG and i.t. injection of TNF-α and IL-1β stimulates CX_3_CL1 mRNA expression in the ipsilateral DRG and in the spinal cord

**Fig. 8:**
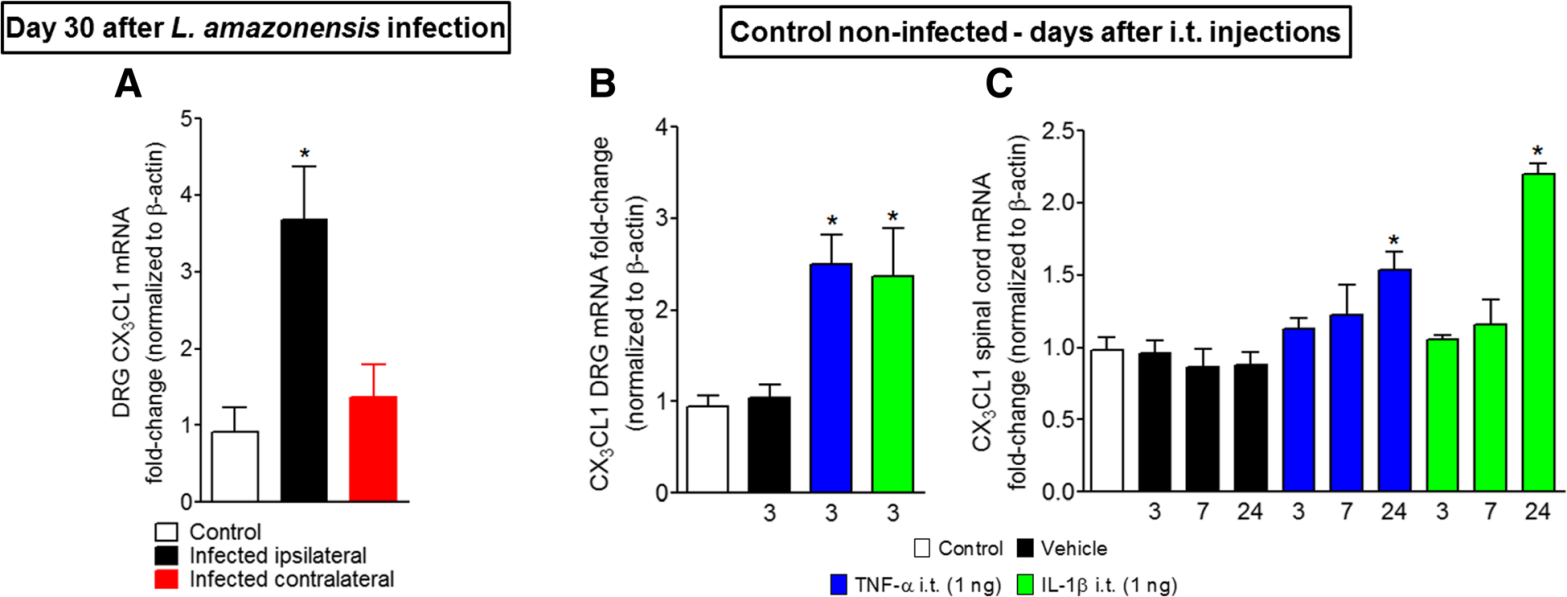
*L. amazonensis* i.pl. infection induces CX_3_CL1 mRNA expression in the ipsilateral DRG, and i.t. injection of TNF-α and IL-1β induces CX_3_CL1 mRNA expression in the ipsilateral DRG and spinal cord. CX_3_CL1 mRNA expression was determined in control non-infected and bilaterally in infected mouse on the 30th day after the infection by RT-qPCR (**a**). **b**, **c** The CX_3_CL1 mRNA expression in DRG (3 h) and spinal cord (3–24 h) after i.t. injections of TNF-α and IL-1β in naïve non-infected animals. Results are presented as mean ± SEM of six mice per group per experiment and are representative of two separated experiments for **a**, **b**, and **c**. *p < 0.05 compared to control non-infected mice (one-way ANOVA followed by Tukey post hoc)

To determine the mechanism of CX_3_CL1 upregulation in the spinal cord of *L. amazonensis*-infected mice, we investigated firstly the CX_3_CL1 mRNA expression in ipsilateral and contralateral DRG of infected mice to determine in which side there would be upregulation. An increase of CX_3_CL1 mRNA expression was observed in the ipsilateral DRG only (Fig. 8a). Considering that inhibition of glial cells reduced TNF-α and IL-1β mRNA expression (Fig. 7b, d), we tested whether these cytokines would represent a possible mechanism of induction of CX_3_CL1 mRNA expression in the spinal cord. TNF-α and IL-1β were injected by i.t. route (1 ng for both) in naïve non-infected mice, and CX_3_CL1 mRNA expression was evaluated by RT-qPCR. CX_3_CL1 mRNA expression was observed in the DRG as early as 3 h after cytokine injection and after 24 h in the spinal cord (Figs. 8b, c). Together, these data suggest a mechanism involving spinal TNF-α and IL-1β in CX_3_CL1 production in the spinal cord after *L. amazonensis* peripheral infection. This effect may represent a positive feedback loop in which the production of TNF-α and IL-1β by glia stimulates the release of CX_3_CL1 by neurons and astrocytes [17, 33] that perpetuate the cycle by the activation of microglia via CX_3_CR1 and, consequently, more TNF-α and IL-1β release.

### Targeting spinal cord astrocytes and microglia inhibits *L. amazonensis*-induced spinal cord NFκB activation

**Fig. 9:**
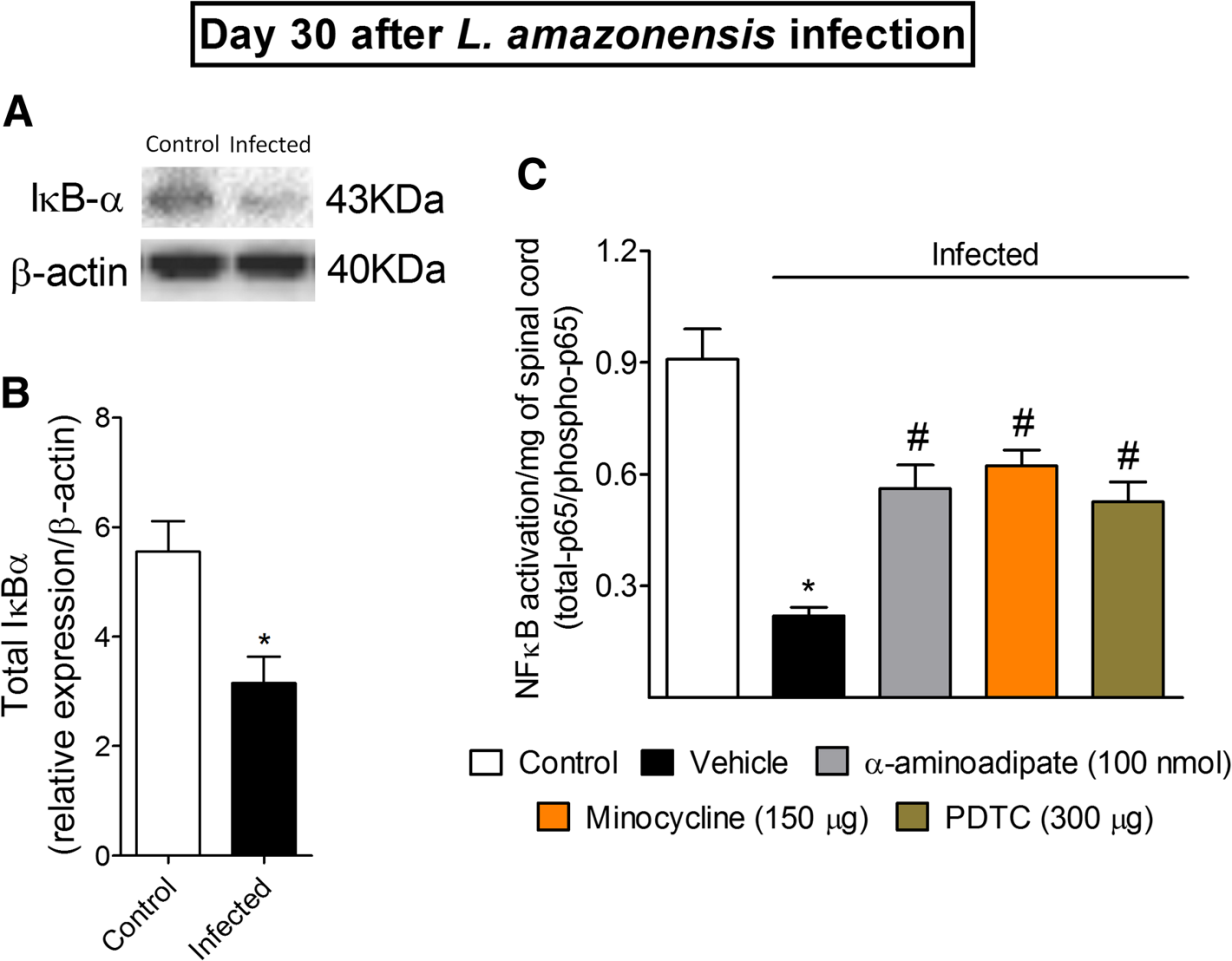
*L. amazonensis* i.pl. infection decreases spinal cord total IκBα expression, and α-aminoadipate, minocycline, and PDTC i.t. treatments inhibit *L. amazonensis*-induced spinal cord NFκB activation. On day 30 after the infection, spinal cord total IκBα protein expression was evaluated by Western blot (four mice per group, **a** and **b**), and NFκB activation was evaluated in control non-infected and infected mice 7 h after treatments with vehicle, α-aminoadipate, minocycline, or PDTC by ELISA (**c**). Results are presented as mean ± SEM of four mice per group per experiment and are representative of two separate experiments for **a** and **b**. For **c**, results are presented as mean ± SEM of six mice per group and are representative of two separated experiments. *p < 0.05 compared to control non-infected mice; ^#^*p* < 0.05 compared to vehicle treated-infected mice (one-way ANOVA followed by Tukey post hoc)

Spinal cord NFκB has a key role in *L. amazonensis*-induced hyperalgesia [2]. Figure 7 shows that the inhibition of glial cells diminishes the mRNA expression of hyperalgesic molecules similarly to NFκB inhibition. Therefore, we investigated the activation of spinal cord NFκB in control non-infected and infected animals as well as whether targeting spinal cord glial cells would also result in NFκB inhibition using Western blot and ELISA assays (Fig. 9). Western blot analysis demonstrated that in control non-infected mice, total IκBα expression is higher when compared to spinal cord samples of infected mice (*p* < 0.05, Fig. 9a). Since IκBα has the function of inhibiting NFκB in the cytoplasm, these data suggest that infection induces NFκB activation. Next, mice were treated by i.t. route with α-aminoadipate and minocycline at the 30th day of *L. amazonensis* infection, and spinal cord samples were collected 7 h after treatment and processed for ELISA (Fig. 9b). The inhibition of astrocytes and microglia diminished the NFκB activation as observed by an increase in total NFκB p65 subunit/phosphorylated NFκB p65 subunit ratio (*p* < 0.05, Fig. 9b). As we have previously observed [2], the control i.t. treatment with PDTC diminished leishmaniasis-induced spinal cord activation of NFκB (*p* < 0.05, Fig. 8b). These results indicate that the activity of glial cells involve the activation of NFκB in the spinal cord during leishmaniasis, which likely explains why targeting glial cells reduces mechanical hyperalgesia, thermal hyperalgesia, and the mRNA expression of hyperalgesic molecules.

## Discussion

Pathophysiological features of pain processing in neglected parasitic infectious diseases such as leishmaniasis remain poorly investigated. The idea that cutaneous leishmaniasis induces painless ulcers likely dampened studying pain in leishmaniasis. However, it is striking that animal models show clear nociceptive behavior [3, 12, 13]. Further, growing body of clinical evidence supports that pain is a symptom of leishmaniasis independently of the region of the body [1, 5–11]. This compelling evidence of pain highlights the importance of understanding the nociceptive mechanisms of leishmaniasis to improve the treatment of pain in human leishmaniasis.

The relation between viral or bacterial infections and glial activation during pain processing was previously investigated using disease models and in vitro analysis [34–36]. CL models show similarities to the human disease; however, immunity, disease outcome, parasite strains, and mice strains limit the homogeneity of data. To the best of our knowledge, this is the first study demonstrating that infection with *L. amazonensis* induces spinal cord neuroinflammation dependent on glia activation that accounts to hyperalgesia. The choice of using only male mice was based on previous demonstration that distinct cell populations in the spinal cord are responsible in mediating mechanical hypersensitivity depending on the gender [20, 21] and also because glial inhibitor minocycline has no effect in female mice regarding nociception [20].

*L. amazonensis* i.pl. infection induces prolonged mechanical and thermal hyperalgesia in mice in the ipsilateral side of the infection, which corroborates our previous results [2] and others using *L. major* infection [3, 12, 13]. Interestingly, we observed that *L. amazonensis* also induced prolonged mechanical hyperalgesia in the contralateral side of the infection, although less intense in comparison with the ipsilateral side. The induction of thermal hyperalgesia was observed in the ipsilateral paw, but not in the contralateral paw to *Leishmania* infection. It is also possible that the neuroinflammatory plastic changes in the spinal cord are responsible for this selective sparing of the contralateral side to sensitization to thermal stimulus by yet undetermined mechanisms [15, 35]. For instance, in a model of CFA-induced masseter muscle inflammation in rats, unilateral injection of CFA caused bilateral allodynia and increased TRPV1 expression in the ipsilateral trigeminal ganglia, but not in the contralateral trigeminal ganglia [37]. The intraplantar injection of CFA also causes bilateral mechanical hyperalgesia and thermal hyperalgesia in the ipsilateral side only [38]. Thus, stimulus was not capable of inducing the same alterations in the contralateral side as in the ipsilateral side to the stimulus. No ulcerative lesions were observed during the experiments, which are important to allow studying the nociceptive phase of disease [2, 9, 13]. Furthermore, *L. amazonensis* infection did not alter ipsilateral ATF3 mRNA expression in DRG cells at the 30th day post-infection indicating no neuronal lesion in contrast with neuropathic pain conditions such as CCI of the sciatic nerve.

*L. amazonensis* induced an increase of MPO activity in the ipsilateral side of the infection indicating neutrophil/monocyte recruitment. TNF-α and IL-1β protein levels also increased in the ipsilateral paw skin tissue at the 40th day of infection, which were not observed in the contralateral non-infected paw skin. Blood TNF-α and IL-1β levels did not change during the course of infection, which suggests that systemic inflammation does not explain the neuroinflammatory events observed in the spinal cord. On the other hand, resident and recruited innate immune cells produce TNF-α and IL-1β at the site of infection [14, 39], which contribute to host protection and killing mechanisms in *Leishmania* infection [40, 41] as well as may account to nociception [42–45]. In fact, it is rational to suppose that TNF-α and IL-1β produced at the infection primary foci explain the hyperalgesia observed before spinal cord glia activation. TNF-α and IL-1β could sensitize nociceptor neurons at an early stage leading to later spinal cord processing of nociceptive information and activation of glial cells that would boost nociception by releasing additional hyperalgesic molecules in the spinal cord [15, 18, 19], therefore contributing to leishmaniasis-induced pain. At the 30th day post-infection, single i.t. treatments with neutralizing anti-CX_3_CL1, etanercept, or IL-1ra inhibited hyperalgesia but not paw edema, which is in line with the notion that peripheral inputs can induce spinal cord neuroinflammation that will enhance nociception [15, 18, 19].

The immunofluorescence data demonstrate *L. amazonensis*-induced bilateral dorsal horn astrocyte and microglia activation at the 30th day post-infection. In pain models in which the injury is unilateral such as spinal nerve ligation (SNL), bilateral detection of glial activity is not observed for up to 28 days after SNL [46]. Although the injection of parasite was performed in only one paw, the parasite may spread and infect other tissues. Time-dependent increase of parasitism was detected only in the ipsilateral draining lymph node. Parasites were also detected in the contralateral lymph node and spleen; however, the parasite load remained stable until the 40th day post-infection. In agreement with our data, evidence supports that the main organs affected up to 90 days post-infection are paw tissue (site of the inoculation of the parasite) and ipsilateral draining lymph node in leishmaniasis [23]. These results suggest that parasite load parallels, at least in part, the observed pain responses [2]. Corroborating this rationale, parasite load increases over time in the draining lymph node of the ipsilateral paw with detectable parasitism starting at the 5th day and increasing up to the 40th day. The immune cellular/molecular events in periphery contribute to produce neuroplasticity in the spinal cord and persistent pain, which might account for long-lasting bilateral pain detected here [15]. Depending on the intensity of local peripheral activation of nociceptor neurons, spinal cord activation in the ipsilateral side can spread to the contralateral side [15]. In the present experimental condition, this is likely to be occurring considering there was minor mechanical hyperalgesia and no thermal hyperalgesia in the contralateral paw, which should be comparable to the ipsilateral side in case of infection spread.

Astrocytes are distributed in the CNS with low levels of GFAP expression and in non-overlapping and organized manner with only the individual most distal tips interdigitation with one another in grey matter. Diseases of the CNS, nerve lesion, activated microglia, cell damage, ischemia, and neuronal hyperactivity can induce astrogliosis, which encompasses molecular and functional changes resulting in progressive modifications ranging from hypertrophy to proliferation and scar formation, and overlapping individual fields. In moderate astrogliosis, there is hypertrophy, increase of GFAP expression, but the individual astrocyte domains are preserved and there is no pronounced overlapping of processes. In severe astrogliosis, there is hypertrophy with high increase of GFAP expression together with proliferation and overlapping of processes [47]. *L. amazonensis* induced a moderate astrogliosis at the 30th day of infection as observed by increase of GFAP expression, hypertrophy, and no pronounced overlapping of astrocyte processes. Upon a pathological event, microglia migrate and proliferate with increasing Iba-1 expression, transform morphologically into more branched and ramified cells than in the resting state, and expand their surveillance area invading the territory of other microglial cells that normally occupy defined territories [48]. The expression of CX_3_CR1 by microglia is also a critical step to the activation of these cells [15]. These phenotypic changes characteristic of microgliosis also occurred in *L. amazonensis* infection.

Spinal cord astrocytes and microglia are described as key cells in the mechanism of pain processing in inflammatory, neuropathic, and cancer models [15, 16, 18, 19]. α-Aminoadipate and minocycline are effective inhibitors used in models of glial cell-dependent chronic pain such as SNL [49, 50]. In the present study, α-aminoadipate and minocycline reduced leishmaniasis-induced ipsilateral mechanical hyperalgesia and thermal hyperalgesia and also contralateral mechanical hyperalgesia. Thus, spinal astrocytes and microglia contribute to *L. amazonensis*-induced hyperalgesia. However, the role of spinal cord glial cells in regulating leishmaniasis-induced hyperalgesia does not seem to occur at all time points of infection since GFAP and Iba-1 mRNA expression peaked at the 30th day of infection. At this day, single i.t. treatment with α-aminoadipate or minocycline inhibited the ipsilateral mechanical and thermal hyperalgesia for up to 24 h. The inhibitory effect of α-aminoadipate on mechanical hyperalgesia lasted up to 48 h and was longer than its effect on thermal hyperalgesia. The inhibition profile of contralateral mechanical hyperalgesia by α-aminoadipate and minocycline occurs between 3 and 48 h after the treatment for both drugs. These behavioral data together with immunofluorescence data suggest a role for activated contralateral astrocytes and microglia in contralateral mechanical hyperalgesia by 30 days of infection. However, contra-lateral mechanical hyperalgesia starts by 10 days of infection, which suggests that mechanisms not addressed in the present study might account to hyperalgesia at earlier stages. It is noteworthy to mention there is evidence that minocycline may induce direct neuronal effects [51, 52]. Minocycline inhibits Na^+^ currents in DRG cells [52] and increases the frequency but not the amplitude of spontaneous inhibitory postsynaptic currents in rat spinal cord substantia gelatinosa neurons in a concentration-dependent manner. These mechanisms might contribute to reduce the excitability of spinal cord neurons and consequently central sensitization [51]. Therefore, minocycline induces analgesia through regulating molecular mechanisms in sensory neurons and inhibiting spinal cord microglia [52]. Nevertheless, we also observed microglia activation, which is a recognized target that leads to analgesia [16]. Thus, minocycline could be targeting neurons and microglia. We avoided using intrathecal cannula for prolonged treatment since this procedure induces local inflammation that is sensible to indomethacin treatment [53], which therefore adds the participation of prostanoids related to the cannula and not the infection itself.

In neuropathic pain, neuronal-glial interactions lead to central sensitization by mechanisms involving the release of primary sensory neuron-derived CX_3_CL1, which binds to its receptor CX_3_CR1 expressed by microglia and activates these cells. In turn, microglia produce hyperalgesic mediators such as TNF-α and IL-1β that together with other pro-nociceptive molecules activate and sensitize spinal cord nociceptor neurons. Astrocytes are also involved in the mechanism of central sensitization and under TNF-α-dependent activation represent important sources of glutamate that further contributes to activate second-order neurons [17, 18]. CX_3_CL1 is described as a key chemokine for enhanced nociceptive response in the spinal cord with increased protein and mRNA expression [17, 54–56]. Herein, the inhibition of leishmaniasis-induced activation of astrocytes, microglia, and NFκB reduced spinal cord CX_3_CL1 mRNA expression as well as *Leishmania* infection induces CX_3_CL1 mRNA expression in the ipsilateral DRG of infected mice. Accordingly, using naïve non-infected mice, we demonstrated that the i.t. injection of hyperalgesic doses of TNF-α and IL-1β [16] stimulate CX_3_CL1 mRNA expression in the spinal cord, which corroborates previous in vitro data using human astrocytes [57]. Although CX_3_CL1 may be induced in spinal cord astrocytes, it is mainly constitutively expressed by DRG neurons and their projections into the spinal cord. Its receptor, CX_3_CR1, may be found in peri-neuronal glia in DRG, but it is primarily expressed by spinal cord dorsal horn microglia with a prominent upregulation in these glial cells in neuropathic pain models, suggesting a neuronal-microglial signaling in spinal cord upon pathological conditions [17, 33, 58]. Spinal cord CX_3_CR1 mRNA expression was detected between 20 and 30 days post-*L. amazonensis* infection, which is rational since its expression is necessary for microglial activation that was showed to start at day 30 [15]. Evidence in other models of pain observed CX_3_CL1 expression by astrocytes and neurons in the spinal cord although the neuronal expression is more widely demonstrated [15, 17, 33, 59]. TNF-α and IL-1β also induced CX_3_CL1 mRNA expression, and glial inhibition reduced TNF-α and IL-1β production, thus suggesting that glial cells regulate CX_3_CL1 mRNA expression via, at least in part, TNF-α and IL-1β during *L. amazonensis* infection. Another possible mechanism contributing to hyperalgesia is the retrograde axonal transport of TNF-α and its receptors TNFR1 and TNFR2 from the periphery to the spinal cord [60]. At the 30th day of *L. amazonensis* infection, spinal TNF-α, TNFR1, and TNFR2 are increased and inhibiting TNF-α reduces the hyperalgesia [2]. However, to our knowledge, it has not been investigated whether retrograde axonal transport of TNF-α and its receptors occurs in leishmaniasis. Nociceptor neurons are sensors of TNF-α and IL-1β [42, 56, 61–63]. *L. amazonensis*-induced pain was inhibited by targeting spinal CX_3_CL1, TNF-α, and IL-1β with pharmacological tools as well as *Leishmania* infection induced an increase of TNF-α, IL-1β, CX_3_CR1, and CX_3_CL1 mRNA expression in the spinal cord, which were inhibited by targeting spinal cord astrocytes, microglia, and NFκB.

The role of spinal cord NFκB in nociception is well documented [64–67]. We have previously demonstrated that spinal cord NFκB is a key transcription factor in pain processing in *L. amazonensis* infection [2]. The present data corroborates it since PDTC i.t. injection inhibited *L. amazonensis*-induced spinal NFkB activation [2]. Nevertheless, we advance by demonstrating that i.t. treatment with inhibitors of astrocytes and microglia reduces NFκB activation, demonstrating that the activity of these cells is related to NFκB activation. NFκB is responsive to TNF-α and IL-1β signaling, and in the opposing direction, these cytokines are NFκB downstream targets [68]. Activated microglia produce large amounts of TNF-α and IL-1β, which in turn sensitize spinal cord nociceptor neurons [15, 18]. Thus, the detection of increased spinal TNF-α and IL-1β mRNA expression and NFκB activation together with hyperalgesia in *L. amazonensis*-infected animals is coherent.

## Conclusions

**Fig. 10:**
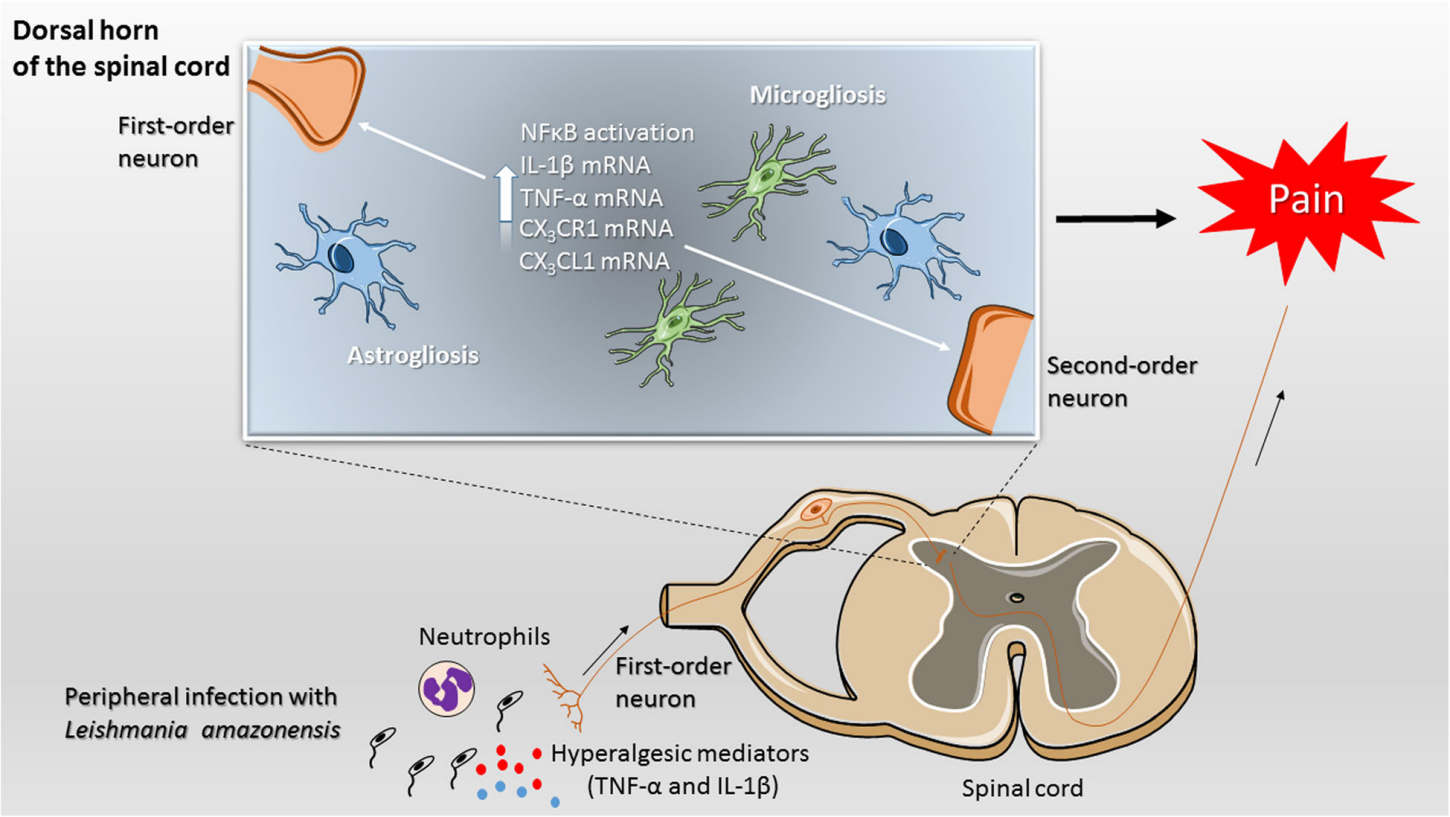
Schematic proposition of the mechanisms involved in *L. amazonensis* infection-induced spinal cord events leading to hyperalgesia in BALB/c mice. The peripheral infection with *L. amazonensis* leads to an immune reaction in infected paw tissue characterized by neutrophil recruitment (increased MPO activity) and elevated production of pro-inflammatory cytokines TNF-α and IL-1β at the primary infection focus, which are well-known sensitizers of first-order neurons. Nociceptive signaling reaches the dorsal horn of spinal cord resulting in increased mRNA expression of CX_3_CL1, CX_3_CR1, microglia, and astrocytes activation and consequent NFκB-dependent TNF-α and IL-1β increased mRNA expression. These pathological modifications in spinal cord sites in response to *L. amazonensis* peripheral infection contributes therefore to the sensitization of first- and second-order neurons and maintenance of a hyperalgesic state in experimental leishmaniasis-induced pain

The present findings reveal a previously unrecognized contribution of spinal cord astrocytes and microglia in *L. amazonensis*-induced hyperalgesia in BALB/c mice. However, we must highlight that this spinal cord neuroinflammation mechanism is not the solely responsible for hyperalgesia in leishmaniasis. Peripheral inflammation in the primary inflammatory foci has certainly an important role in pain. Parasite burden in the paw and draining lymph node parallels hyperalgesia depending on the time point, which together with evidence that some virulence factors can activate nociceptor sensory neurons [35, 36], suggests this is also a potential mechanism. There is a crosstalk between neurons and glial cells in the spinal cord via cytokines and chemokines in a NFκB-dependent manner, which induce nociceptor sensory neurons sensitization in the ipsilateral and contralateral sides relative to infection. Finally, it is likely that in the present experimental condition, leishmaniasis induces an inflammatory pain since there was no detection of altered ATF3 mRNA expression in DRG neurons. Figure 10 presents a schematic conclusion of the present data.

## Additional files

Additional file 1: **Figure S1.** Absence of detection of *L. amazonensis*-induced ATF3 mRNA expression in the DRG. ATF3 mRNA expression in DRG was determined in control non-infected and bilaterally in infected mice at day 30 after the infection by RT-qPCR. CCI ipsilateral control group was included for comparison with the group infected with *L. amazonensis*. Results are presented as mean ± SEM of six mice per group per experiment and are representative of two separate experiments. *p < 0.05 compared to control non-infected mice (one-way ANOVA followed by Tukey post hoc). (TIF 975 kb)

Additional file 2: **Figure S2.** The temporal profile of TNF-α and IL-1β plasmatic levels do not change after i.pl. *L. amazonensis* infection. TNF-α (A) and IL-1β (B) plasmatic levels were determined in control non-infected and infected mice after the infection (5–40 days) by ELISA. Results are presented as mean ± SEM of six mice per group per experiment and are representative of two separated experiments (one-way ANOVA followed by Tukey post hoc). (TIF 533 kb)

Additional file 3: **Figure S3.** Temporal profile of ipsilateral draining lymph node, spleen, and contralateral lymph node parasitism after i.pl. *L. amazonensis* infection. Ipsilateral draining lymph node (A), spleen (B), and contralateral lymph node (C) parasitism were determined after the infection (5–40 days) by real-time qPCR. Results are presented as mean ± SEM of six mice per group per experiment and are representative of two separate experiments. *p < 0.05 compared to the day 0; ^#^*p* < 0.05 compared to the days 0 and 5; ƒ*p* < 0.05 compared to days 0, 5, and 10 (one-way ANOVA followed by Tukey post hoc). The results are expressed as parasites equivalent per 100 ng of *Leishmania* DNA. ND: not detected. (TIF 540 kb)

Additional file 4: **Figure S4.** Detection of spinal cord CX_3_CR1 induced by i.pl. *L. amazonensis* infection. CX_3_CR1 mRNA expression was determined in control non-infected and infected mice after the infection (5–40 days) by RT-qPCR. Results are presented as mean ± SEM of six mice per group per experiment and are representative of two separated experiments for panel. *p < 0.05 compared to control non-infected mice (one-way ANOVA followed by Tukey post hoc). (TIF 696 kb)

Additional file 5: **Figure S5.** α-Aminoadipate and minocycline i.t. treatments inhibits *L. amazonensis*-induced contralateral mechanical hyperalgesia, without inducing effects in contralateral thermal hyperalgesia and paw edema. Contralateral mechanical (A) and thermal (B) hyperalgesia and paw edema (C) were measured in control non-infected and infected mice on day 30 after the infection, and subsequently, infected mice received i.t. injection of α-aminoadipate (selective astrocyte inhibitor, 100 nmol), minocycline (microglia inhibitor, 150 μg), or vehicle for measurement of contralateral mechanical and thermal hyperalgesia and paw edema. Results are presented as mean ± SEM of six mice per group per experiment and are representative of two separate experiments. *p < 0.05 compared to control non-infected mice (one-way ANOVA followed by Tukey post hoc). (TIF 777 kb)

## Abbreviations

CL: Cutaneous leishmaniasis
CNS: Central nervous system
CX_3_CL1: C-X_3_-C motif ligand 1
CX_3_CR1: C-X_3_-C chemokine receptor 1
DRG: Dorsal root ganglia
GFAP: Glial fibrillary acidic protein
Iba-1: Ionized calcium-binding adapter molecule 1
IL-1ra: Interleukin-1 receptor antagonist
IL-1β: Interleukin-1β
IκB-α: Nuclear factor of kappa light polypeptide gene enhancer in B cells inhibitor alpha
MPO: Myeloperoxidase
NFκB: Nuclear factor kappa B
PDTC: Pyrrolidine dithiocarbamate
TNFR2: Tumor necrosis factor receptor 2
TNF-α: Tumor necrosis factor alpha
