# Supplementary figures and images for: Contribution of spinal cord glial cells to L. amazonensis experimental infection-induced pain in BALB/c mice
Source: J Neuroinflammation. 2019 May 28;16:113. doi: 10.1186/s12974-019-1496-2 (PMC6540403; doi:10.1186/s12974-019-1496-2)

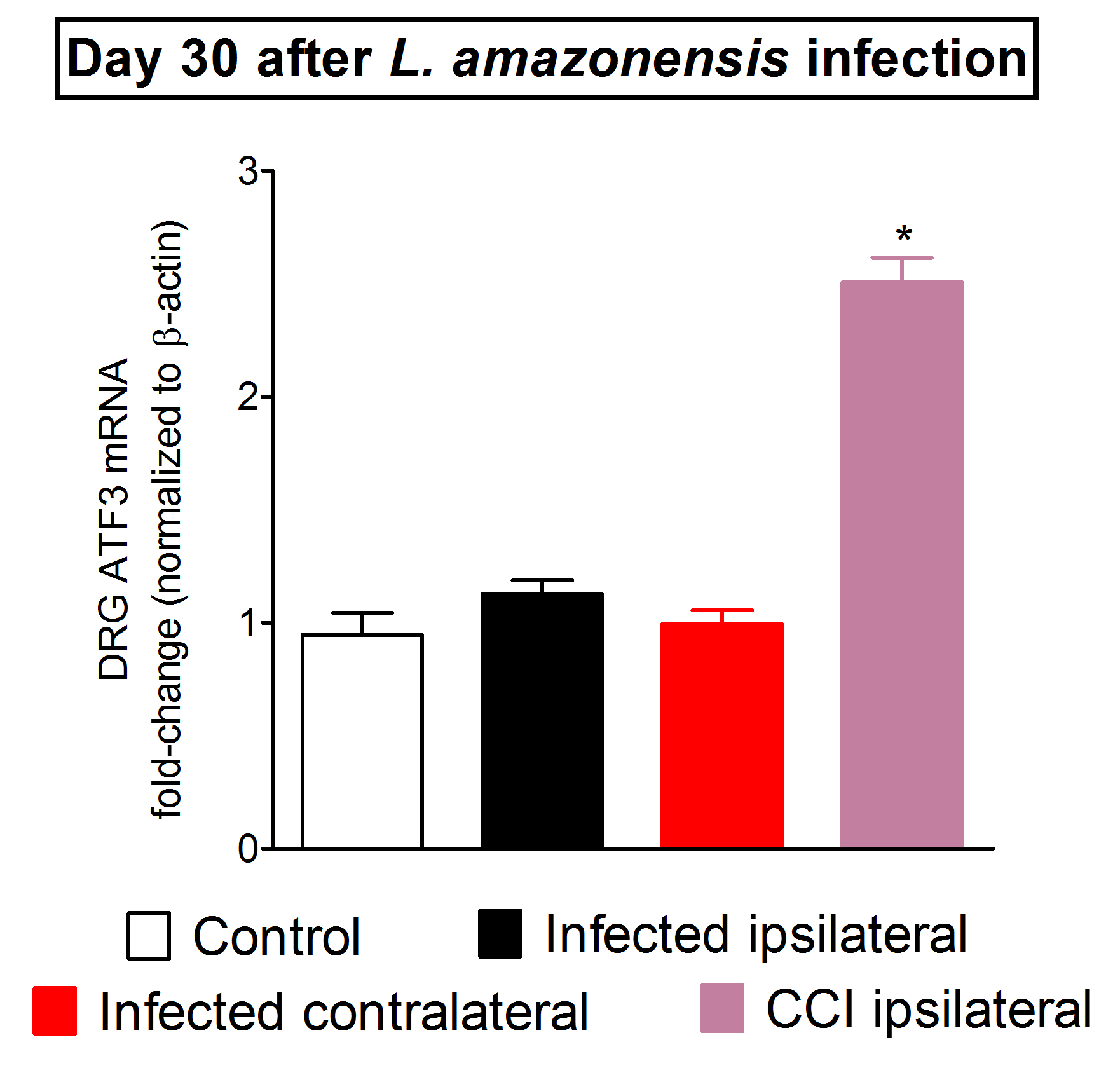

Supplement: Supplementary file 1 — Figure S1. Absence of detection of L. amazonensis-induced ATF3 mRNA expression in the DRG. ATF3 mRNA expression in DRG was determined in control non-infected and bilaterally in infected mice at day 30 after the infection by RT-qPCR. CCI ipsilateral control group was included for comparison with the group infected with L. amazonensis. Results are presented as mean ± SEM of six mice per group per experiment and are representative of two separate experiments. *p < 0.05 compared to control non-infected mice (one-way ANOVA followed by Tukey post hoc). (TIF 975 kb) [file 12974_2019_1496_MOESM1_ESM.tif]

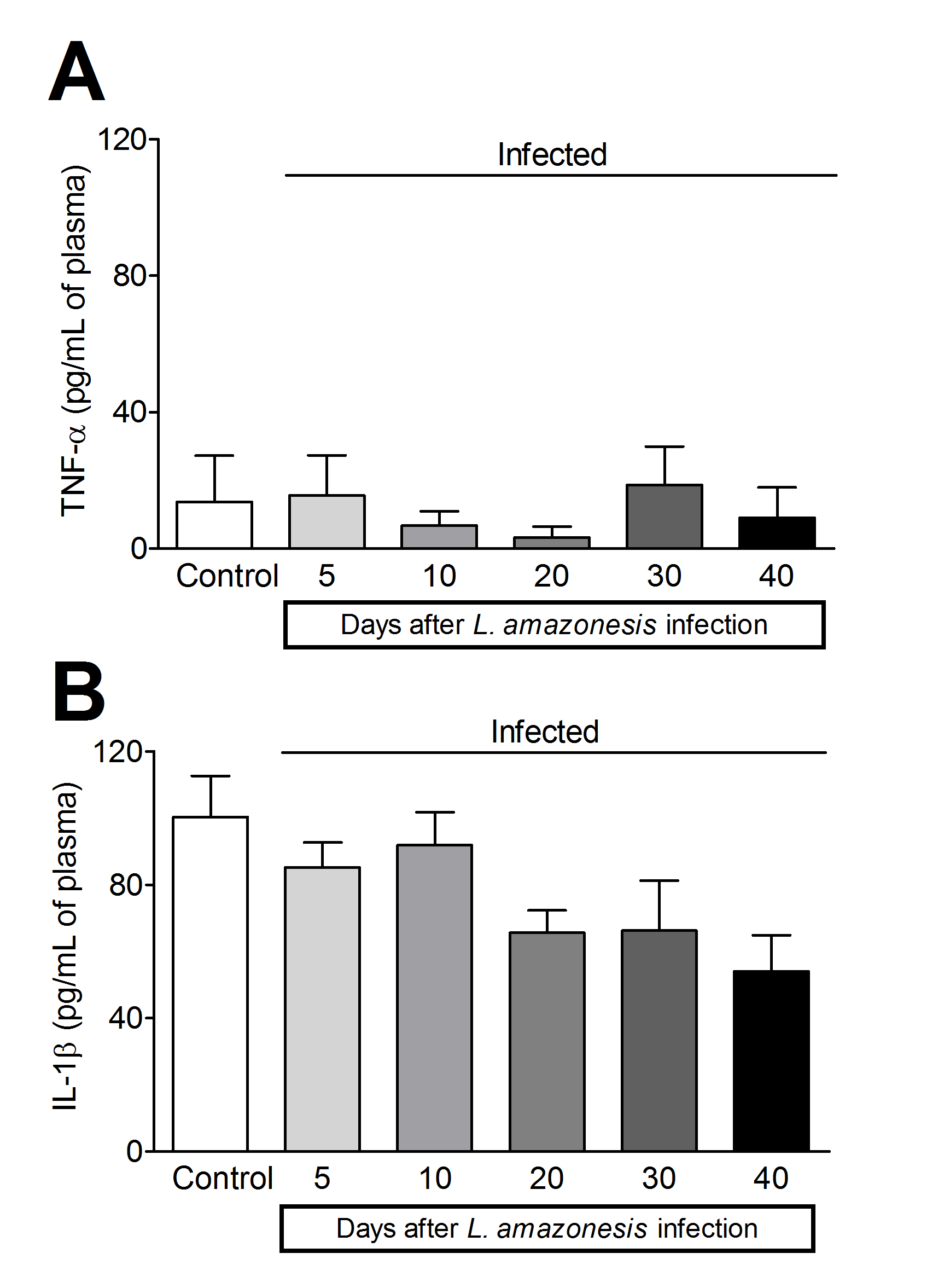

Supplement: Supplementary file 2 — Figure S2. The temporal profile of TNF-α and IL-1β plasmatic levels do not change after i.pl. L. amazonensis infection. TNF-α (A) and IL-1β (B) plasmatic levels were determined in control non-infected and infected mice after the infection (5–40 days) by ELISA. Results are presented as mean ± SEM of six mice per group per experiment and are representative of two separated experiments (one-way ANOVA followed by Tukey post hoc). (TIF 533 kb) [file 12974_2019_1496_MOESM2_ESM.tif]

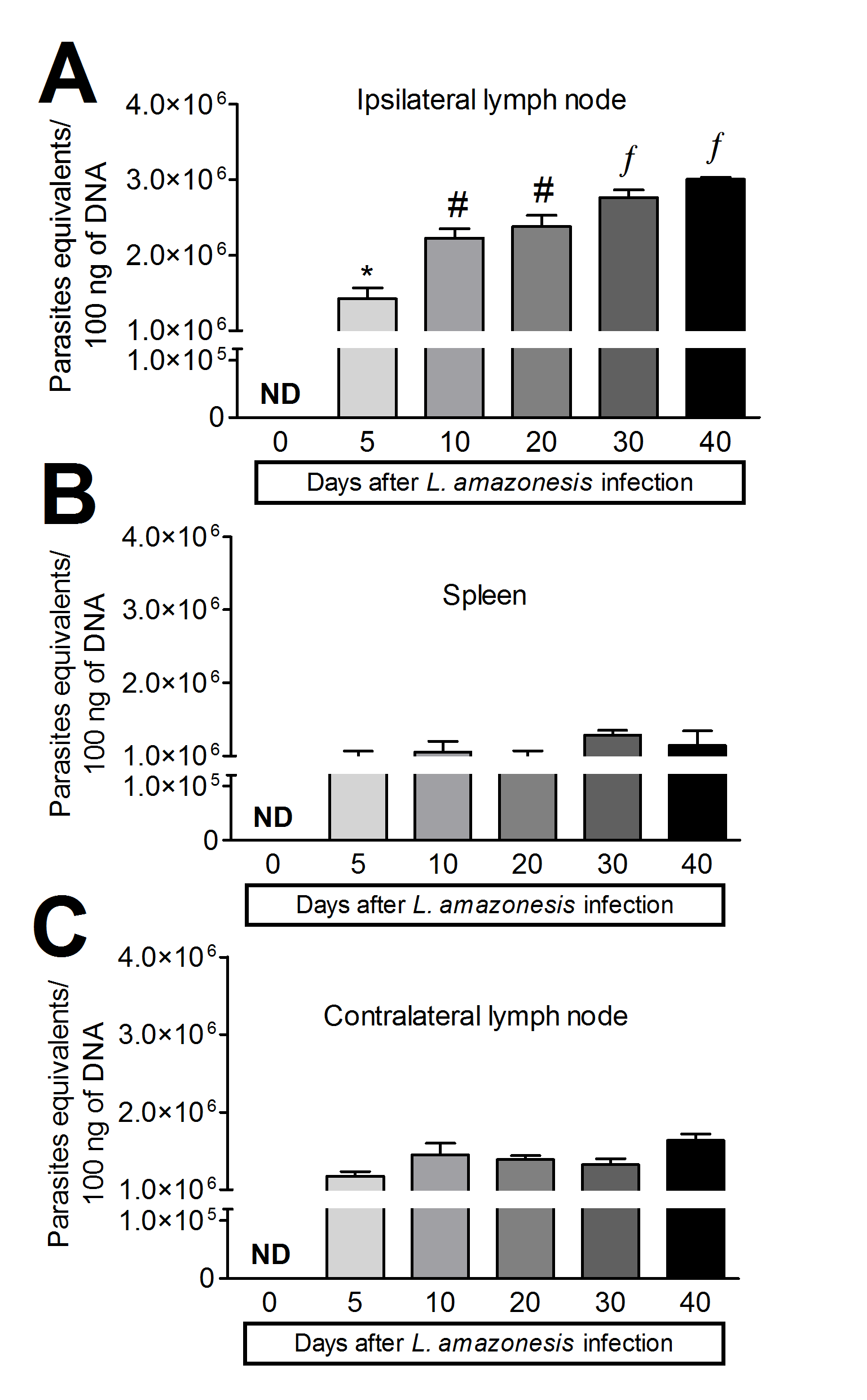

Supplement: Supplementary file 3 — Figure S3. Temporal profile of ipsilateral draining lymph node, spleen, and contralateral lymph node parasitism after i.pl. L. amazonensis infection. Ipsilateral draining lymph node (A), spleen (B), and contralateral lymph node (C) parasitism were determined after the infection (5–40 days) by real-time qPCR. Results are presented as mean ± SEM of six mice per group per experiment and are representative of two separate experiments. *p < 0.05 compared to the day 0; #p < 0.05 compared to the days 0 and 5; ƒp < 0.05 compared to days 0, 5, and 10 (one-way ANOVA followed by Tukey post hoc). The results are expressed as parasites equivalent per 100 ng of Leishmania DNA. ND: not detected. (TIF 540 kb) [file 12974_2019_1496_MOESM3_ESM.tif]

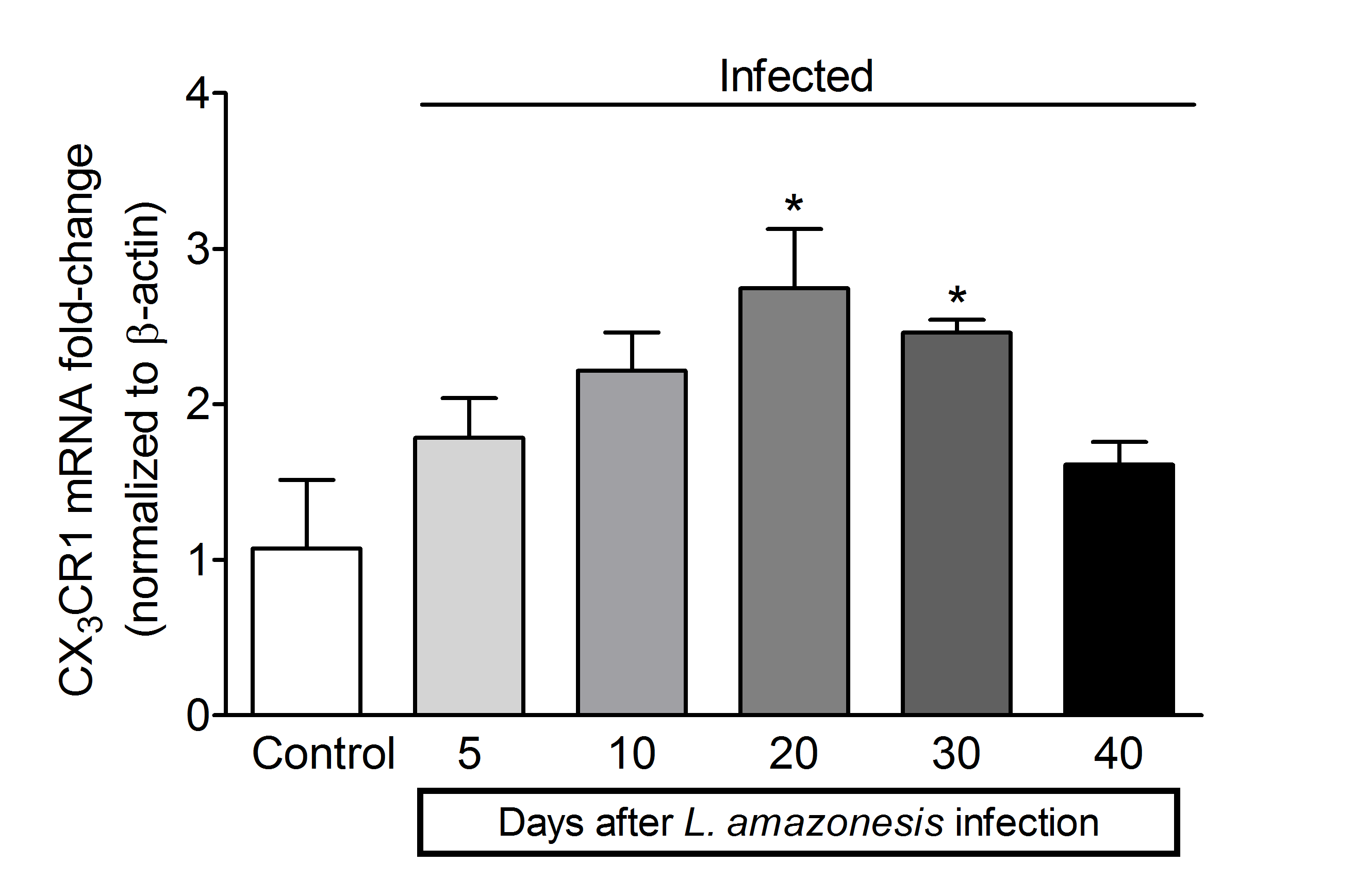

Supplement: Supplementary file 4 — Figure S4. Detection of spinal cord CX3CR1 induced by i.pl. L. amazonensis infection. CX3CR1 mRNA expression was determined in control non-infected and infected mice after the infection (5–40 days) by RT-qPCR. Results are presented as mean ± SEM of six mice per group per experiment and are representative of two separated experiments for panel. *p < 0.05 compared to control non-infected mice (one-way ANOVA followed by Tukey post hoc). (TIF 696 kb) [file 12974_2019_1496_MOESM4_ESM.tif]

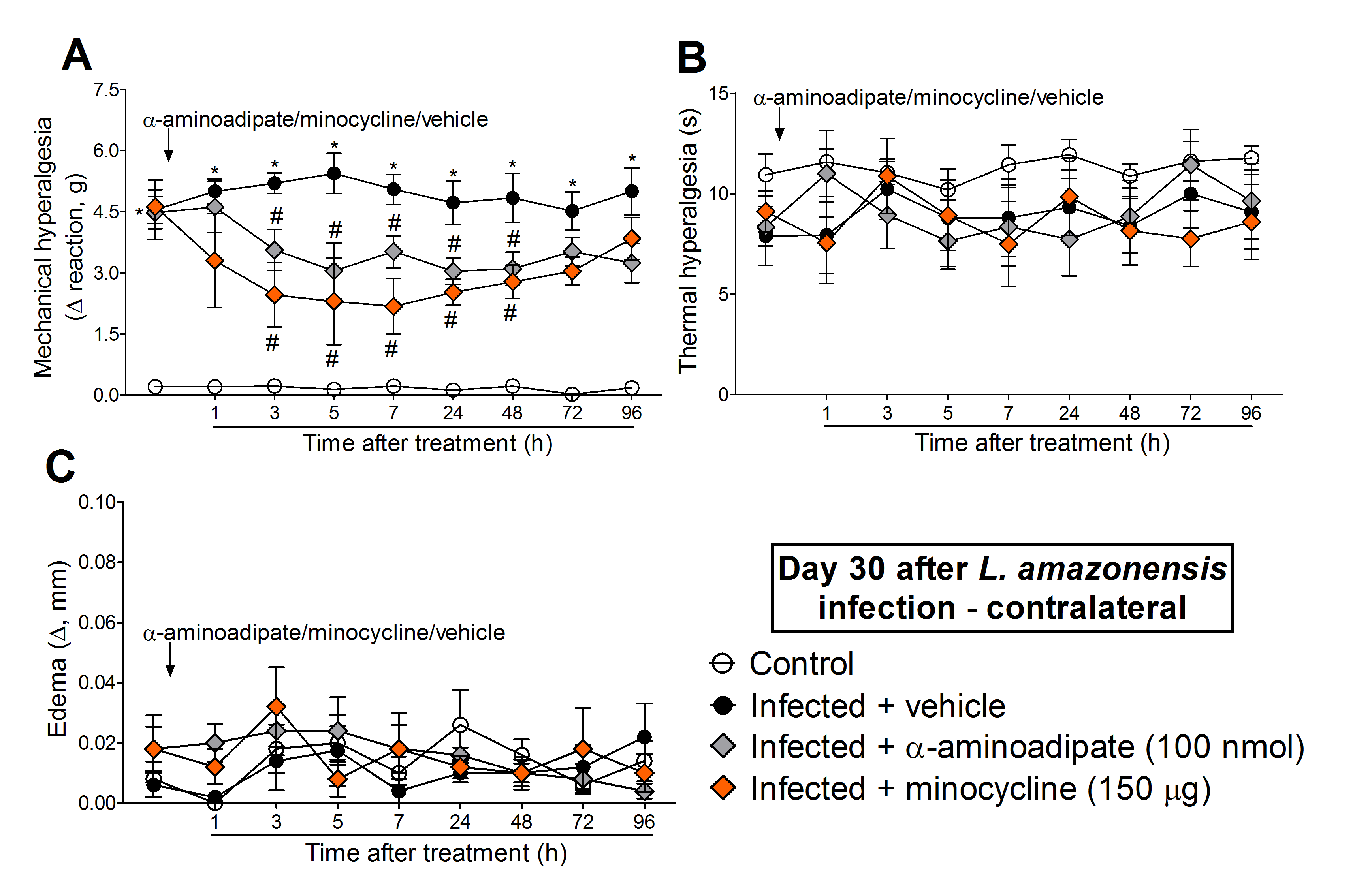

Supplement: Supplementary file 5 — Figure S5. α-Aminoadipate and minocycline i.t. treatments inhibits L. amazonensis-induced contralateral mechanical hyperalgesia, without inducing effects in contralateral thermal hyperalgesia and paw edema. Contralateral mechanical (A) and thermal (B) hyperalgesia and paw edema (C) were measured in control non-infected and infected mice on day 30 after the infection, and subsequently, infected mice received i.t. injection of α-aminoadipate (selective astrocyte inhibitor, 100 nmol), minocycline (microglia inhibitor, 150 μg), or vehicle for measurement of contralateral mechanical and thermal hyperalgesia and paw edema. Results are presented as mean ± SEM of six mice per group per experiment and are representative of two separate experiments. *p < 0.05 compared to control non-infected mice (one-way ANOVA followed by Tukey post hoc). (TIF 777 kb) [file 12974_2019_1496_MOESM5_ESM.tif]
